# Composing a Tumor Specific Bacterial Promoter

**DOI:** 10.1371/journal.pone.0155338

**Published:** 2016-05-12

**Authors:** Igor V. Deyneko, Nadine Kasnitz, Sara Leschner, Siegfried Weiss

**Affiliations:** 1 Molecular Immunology, Helmholtz Centre for Infection Research, Braunschweig, Germany; 2 Institute of Immunology, Medical School Hannover, Hannover, Germany; Virginia Tech University, UNITED STATES

## Abstract

Systemically applied *Salmonella enterica* spp. have been shown to invade and colonize neoplastic tissues where it retards the growth of many tumors. This offers the possibility to use the bacteria as a vehicle for the tumor specific delivery of therapeutic molecules. Specificity of such delivery is solely depending on promoter sequences that control the production of a target molecule. We have established the functional structure of bacterial promoters that are transcriptionally active exclusively in tumor tissues after systemic application. We observed that the specific transcriptional activation is accomplished by a combination of a weak basal promoter and a strong FNR binding site. This represents a minimal set of control elements required for such activation. In natural promoters, additional DNA remodeling elements are found that alter the level of transcription quantitatively. Inefficiency of the basal promoter ensures the absence of transcription outside tumors. As a proof of concept, we compiled an artificial promoter sequence from individual motifs representing FNR and basal promoter and showed specific activation in a tumor microenvironment. Our results open possibilities for the generation of promoters with an adjusted level of expression of target proteins in particular for applications in bacterial tumor therapy.

## Introduction

Cancer is one of the most frequent cause of death and its incidence is rising [1]. This renders the development of powerful therapeutic strategies of high demand. Besides the improvement of established treatment schedules, alternative therapies need to be exploited to eventually win the fight against this disease. One of such non-conventional strategies that is presently intensively followed, is bacteria-mediated tumor therapy [2]. Several preclinical and clinical trials have been initiated along this line [3–6]. The approach is based on an observation that cancer patients with bacterial infections sometimes experience spontaneous regression of their tumor [7]. In the meantime, it was shown for several kinds of bacteria that they are able to target and colonize solid tumors after systemic administration [2]. Apart from obligate anaerobic bacteria like *Clostridia* spp. that are able to grow exclusively in necrotic tumor areas without oxygen supply, facultative anaerobes like *Salmonella enterica* spp. have been shown to target tumors and spread throughout the entire neoplastic tissue.

Besides their inherent anti-cancer effect, these bacteria offer the possibility to act as transport vehicles for therapeutic agents. Such molecules are usually toxic and should exclusively be expressed directly in the tumor. On the other hand, to be most effective, sufficient concentrations of such therapeutic molecules should be reached throughout the entire cancerous tissue. Facultative anaerobic bacteria like *Salmonella* would be perfect candidates to be used as such transporters. However, the normal target organs of *Salmonella*, spleen and liver, are also colonized in tumor bearing hosts. Thus, to prevent the destruction of these healthy tissues the expression of the therapeutic agent must be exclusively restricted to the tumor mass.

In our previous work [8] we could isolate from the genome of *S*. Typhimurium several DNA fragments containing promoters that specifically respond to the physiological conditions of cancerous tissue (promoters and associated genes are listed in S1 Table). These fragments were classified into groups depending on the level of differential expression in tumor tissue and spleen. The latter served as an example of a normal target organ. First bioinformatics analysis of promoters showing high expression in tumors revealed a so-called *tusp* motif apparently responsible for tumor specific activation [8]. However, further experimental data and advanced bioinformatics analysis of other groups of fragments with lower expression in tumors or with limited expression in spleen revealed that it was an oversimplification [9, 10]. Therefore, it was required to thoroughly investigate the principles of the tumor specific transcriptional regulation and reveal contributing functional elements. Understanding such principles will not only allow the optimization of existing promoters but possibly also the creation of new promoters with the required expression profile and high transcriptional activity. In addition, these promoters can serve as probes to understand specific activating conditions provided by the microenvironment of a solid tumor.

## Results

### Basal promoter elements

**Fig 1:**
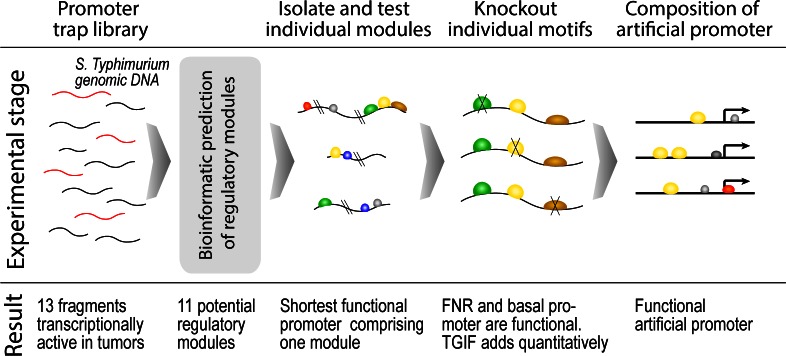
Identification and analysis steps of tumor specific promoters. A schematic overview. Two programs for basal promoter recognition, one based on sequence alignment kernel [11] and another on Hidden Markov Model (HMM) [12] were applied to the TSP set (DNA sequences are given in the S1 Text). As a negative control, a set of DNA fragments that do not initiate expression either in tumor or in spleen was selected (negative promoters, NP). Both programs recognize potential promoters in either dataset and the number of predictions greatly depends on user-defined threshold parameters. To evaluate the specificity of predictions, it was assumed that a basal promoter should be recognized in at least 75% of TSP and at most 50% of NPs (see Methods). This will ensure the generality and specificity of the recognized feature.

The strategy employed here is illustrated in Fig 1. The DNA fragments isolated from the *S*. Typhimurium genome containing promoters with high expression in tumors and absence of expression in spleen and liver were identified using promoter trap library strategies [8]. Based on the fact that *Salmonella* mainly targets these two organs and only traces of the bacteria could be detected in the rest of the body we have defined these fragments as tumor specific promoters or TSP. Fragments were fused with the bare GFP coding DNA sequence preceded only by a ribosomal binding site (Shine-Dalgarno box). Therefore, TSPs should contain basal promoter structures like -10 and -35 elements as a prerequisite for gene transcription.

The kernel method identified basal promoters in 12 out of 13 TSPs giving a frequency of *BasalP*^*TSP*^ = 0.92. In the NP set the frequency is *BasalP*^*NP*^ = 0.27. For HMM, the values are *BasalP*^*TSP*^ = 0.83 and *BasalP*^*NP*^ = 0.43, respectively. It is clear, that the specificity of predictions by the kernel method is much higher than by HMM. Thus, the kernel method was assumed to be more reliable and results obtained by it considered further. To identify exact positions of TATA-box and *Inr* element, program BROM [13] was applied.

### Tumor specific regulatory elements

Tumor specific transcriptional activation is apparently achieved by features encoded in the DNA sequence of TSP promoters. Such features could be, for example, transcription factor binding motifs or specific conformations of the DNA. Therefore, it was hypothesized that TSP promoters should contain a motif or motifs that either activate transcription exclusively in tumors and/or suppress basal promoter activity in tissues other than tumors such that the observed specificity of transcription would be achieved.

The remainder of this subsection will be organized as follows: i) identification of known DNA motifs, ii) identification of novel DNA motifs, iii) identification of other possible features of the promoters and finally iv) combinatorial analysis of identified motifs and other features.

Recognition of known transcription factor binding motifs on DNA is usually carried out with the help of position weight matrixes (PWMs) that are collected in databases like DPInteract [14], TRANSFAC [15] and JASPAR [16]. Although the latter two describe PWMs of eukaryotic transcription factors, they still can be applied to our data, given that the following is kept in mind: a “eukaryotic motif” identified in a prokaryotic genome can be bound by a completely different protein factor. Thereby, the eukaryotic PWM libraries should be regarded, in our case, solely as a library of DNA motifs.

**Fig 2:**
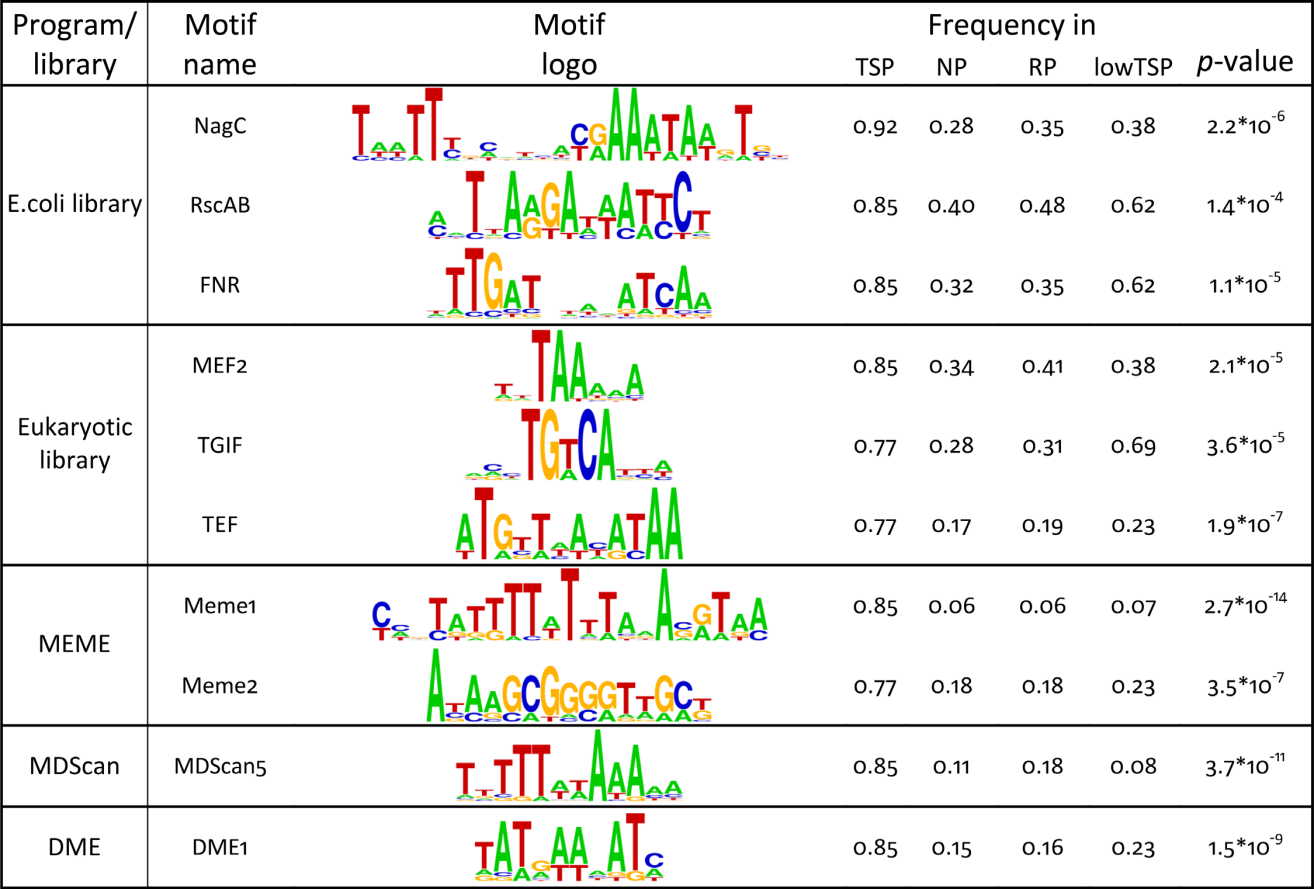
Top motifs identified in the set of tumor specific promoters and its distribution within different groups of promoters. Values are normalized numbers of promoters in a set containing at least one motif. *P*-values were calculated as a binomial probability to observe the actual number of promoters with a motif in the TSP set compared to RP set. A potential overfitting effect of prediction methods can be estimated using an independent lowTSP set (see Methods).

Potential binding motifs were identified using all three databases. Thresholds for each PWM were varied to maximize the discrimination between TSP and NP as described in Methods. Following this strategy, motifs for a number of prokaryotic (DnaA, FNR, NagC and RscAB) and eukaryotic (BRSZ4, HNF1, MEF2, SOX9, TGIF and TEF) transcription factors were identified as specific for the TSP dataset. Top scoring motifs are shown in Fig 2.

Along with library based searches, programs for DNA motif detection exist that do not require databases of PWMs. Such programs evaluate the statistical occurrence of DNA motifs and usually do not differentiate between pro- and eukaryotic genomes. Several programs were applied to our data and the resulting PWMs were examined for specificity to the TSP set as above. Only a few programs identified specific motifs, namely: 6 motifs by Meme [17], 2 by DME [18], 2 by CMF [19] and 5 by MDScan [20]. Given that these programs do not suggest any biological function of the recognized motifs, we named these motifs by program name followed by a number. Most significant motifs are included in Fig 2 and a full list is given in S1 Fig.

Features like nucleotide composition are also known to affect gene expression. It was found that TSP promoters are in general AT-rich (*AT*^*TSP*^ = 0.512, *AT*^*NP*^ = 0.468) and can be specifically characterized by the presence of AT-rich regions (*ATregion*^*TSP*^ = 0.77, *ATregion*^*NP*^ = 0.22, see Methods). In particular, an (A)_8_ repeat is often found in the TSP set (*A*_*8*_^*TSP*^ = 0.77), but not in the set of NP (*A*_*8*_^*NP*^ = 0.47). This feature may represent a general transcriptional activity of the promoters not connected to tumor activation and therefore may represent a general promoter feature.

It is clear from the above that many motifs in the TSP set could be identified. Each might explain to some extent tumor specific transcriptional activity. However, before proceeding to experimental testing of each motif it appeared more efficacious first to search for specific groups of motifs and to test such groups rather than every single motif. Clearly, it would be beneficial if potential groups of motifs would localize densely in the promoters, such that a cut-and-test strategy could be applied.

In general, promoters of genes vary greatly in length. Therefore, no reasonable window size parameter as required for many programs could be suggested for our particular dataset. Therefore, we have developed a bioinformatics method that identifies combinations of heterogeneous features, like, DNA motifs, CpG islands, repeats, that are co-localized on a DNA sequence [10]. This method is based on a genetic algorithm and searches for a collection of motif combinations that exhibit high specificity for the positive dataset and localize separately on DNA sequences.

Using this method several highly specific combinations of DNA motifs were identified (listed in S2 Table). Further it was decided to perform the experiments in two steps. First, promoters P0.48, P0.156, P0.271, P0.272 and P0.301 were split into three fragments such that the 5' and 3' fragments preferably contain a single combination. Here, we denote these promoters as "P0." (round 0) followed by a number corresponding to the number used in [8]. For further identification, names of promoter fragments will be supplemented with an underscore and a consecutive number. By testing such fragments, it became possible to exclude many non-functional motif combinations.

**Fig 3:**
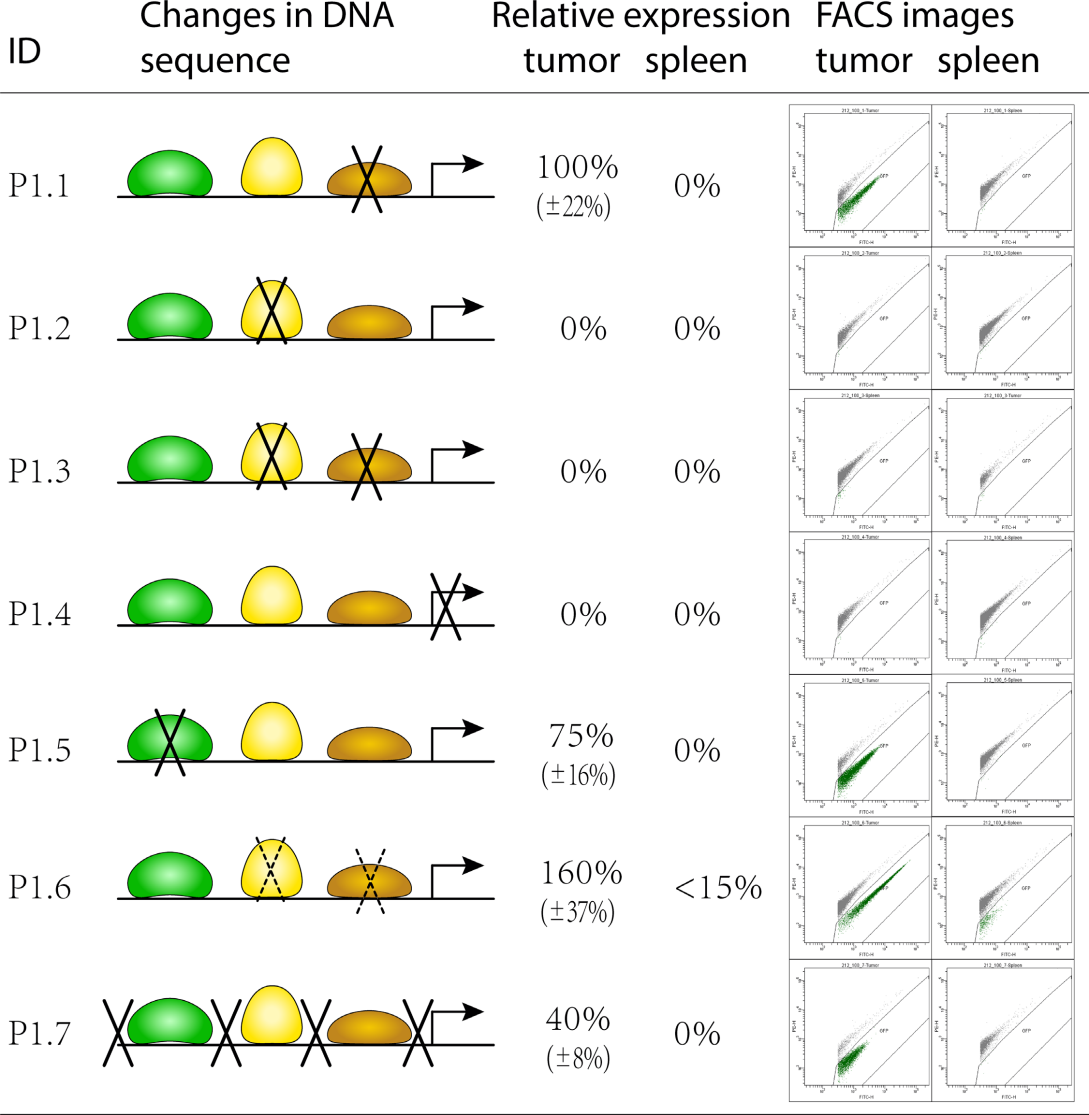
Schematic representation of promoter structure. Left: binding motifs for factors TGIF, FNR and NagC are shown in green, yellow and brown, respectively. Basal promoter is shown as a directed arrow. Knockout of essential nucleotides within motifs were according to the literature and are represented by crossing lines. Mutation of non-essential nucleotides within motifs was random and is represented by dashed crossing lines. Nucleotides outside motifs were mutated randomly. Right: representative flow-cytometric analyses of GFP-expression in tumor and spleen. Each green point on the blots represents GFP expression levels of an individual bacterial cell. Displayed values of expression are relative to the expression values of the original promoter P0.212_1. All nucleotide substitutions are presented in the S2 Fig.

Second, on the basis of localization the remaining motifs, promoters P0.212, P0.134 and several functional fragments from the first step were split into shorter fragments of 50-100bp. As in the previous step, the rationale in selection of fragments is to efficiently separate testable motifs. By experimental analysis the shortest promoter P0.212_1 comprising one functional module was identified (schematically shown in Fig 3). This module consists of three DNA motifs for factors TGIF, FNR and NagC, respectively, complemented by basal promoter elements.

### Functional role of each regulatory element

The promoter model identified above consists of three regulatory DNA elements and according to the literature all of them may regulate transcription both positively and negatively [21–23]. To test the functionality of each element and to establish its contribution to the overall effect, a knock out strategy was implemented. Each motif in P0.212_1 was mutated at positions designated as critical in studies where the motif had been discovered (Fig 3, sequences are given in S2 Fig). In addition, to evaluate the prediction of the basal promoter we mutated the TATA-box by introducing 'G' and 'C' nucleotides. Finally, to verify that there are no other elements which had not been discovered in the previous step, intra-motif spacers were also mutated. All variants of P0.212_1 were synthesized *de novo* (promoters P1.1 –P1.7, here and further named as round 1 promoters "P1."), cloned and confirmed by sequencing. Results of the analysis by flow cytometry are presented in Fig 3.

From this analysis it became clear that the motif for transcription factor NagC is not functional. Mutation of this motif did not change differential tumor specific expression. On the other hand, modification of the FNR motif completely disrupted transcription (clone P1.2, FNR knockout). The same effect was found for the TATA-box (clone P1.4, TATA knockout). Deletion in the TGIF motif reduced transcription to approx. 75% of the level of the original promoter (clone P1.5, TGIF knockout). Mutations at insignificant positions in motifs FNR and NagC (clone P1.6) did not influence specificity of transcription, but led to increase in expression in tumors by approximately 60% (Fig 3). Changes of nucleotides in intra-motif regions did not influence the transcription specificity, but reduced its level down to 40% (clone P1.7).

**Fig 4:**
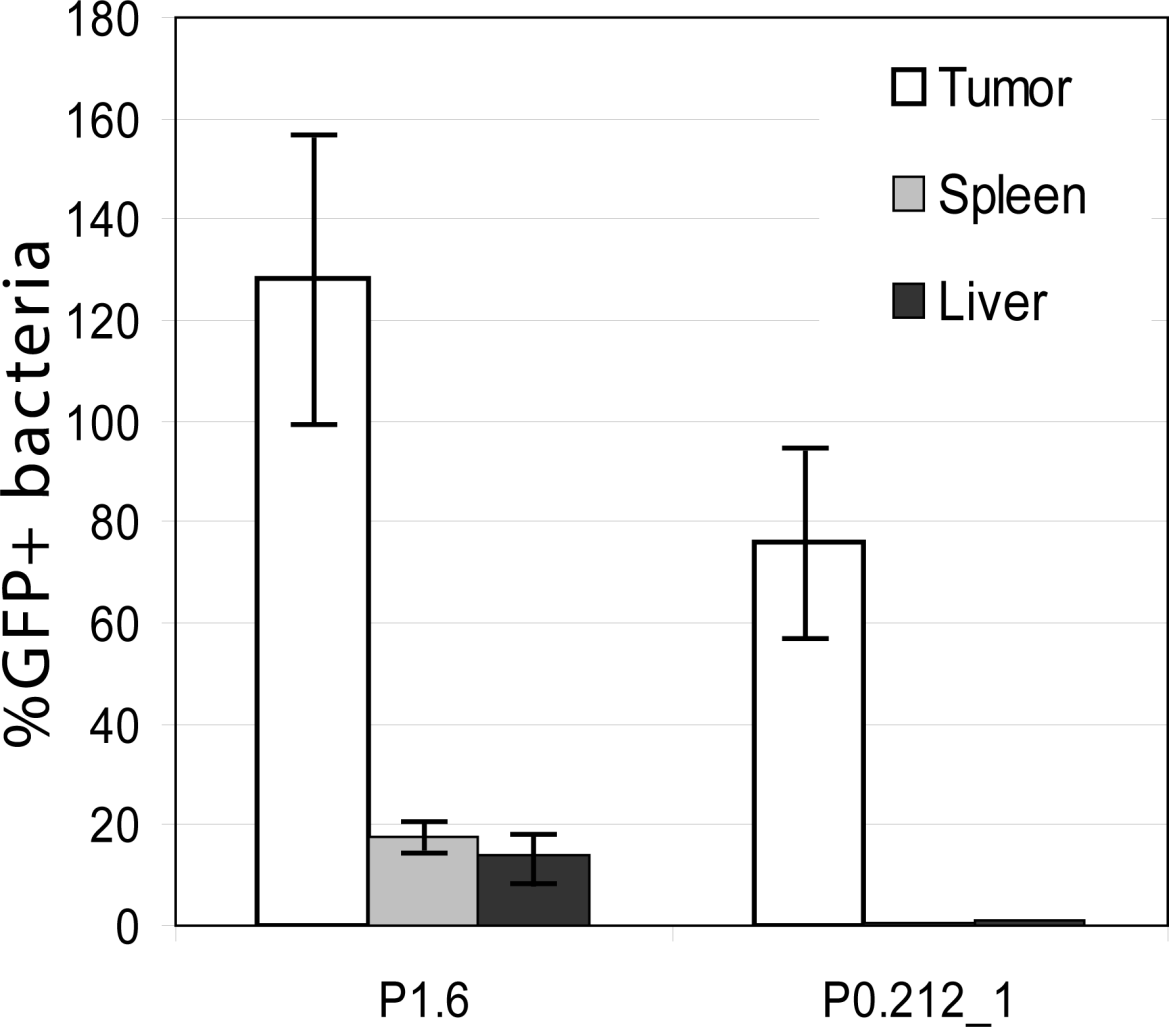
Expression of promoters P1.6 and P0.212_1 in tumor, spleen and liver. Homogenates were analyzed via two color flow cytometry and plating to allow normalization. Given are mean and SD. Expression of P1.6 in spleen and liver can be considered negligible compared to expression in tumor. Therefore expression of promoter P1.6 was accepted as tumor specific.

In all the experiments a unified threshold to separate signal from cellular debris was used. In case of promoter 1.6, this led to detection of a weak GFP signal in spleen (Fig 3). Additional tests including liver as another target organ of *Salmonella* yielded the same results (Fig 4). However, taking into account the enhanced expression in the tumor, the expression in liver and spleen can be considered as negligible.

Taken together: two elements–FNR motif and the basal promoter–form a backbone of a tumor specific bacterial promoter. Other elements, like TGIF and a general nucleotide context, exhibit a minor role and may intensify or downgrade the transcription.

### Artificial promoter constructs

Having identified the principle components of a tumor specific promoter, it was challenging to develop a synthetic promoter with potentially improved characteristics. This would additionally prove the concept of two specific elements that are necessary and sufficient for the tumor specific expression. As a basis for such a promoter, a DNA fragment that cannot initiate any expression was taken from the negative promoters (NP) set. A randomly selected region of 100bp from this fragment was used as template. The idea here was to create a minimal set of promoters comprising all discovered functional elements for FNR and TSS including their consensus sequences from the literature. This should confirm the validity of the proposed principle and the functionality of each element.

**Fig 5:**
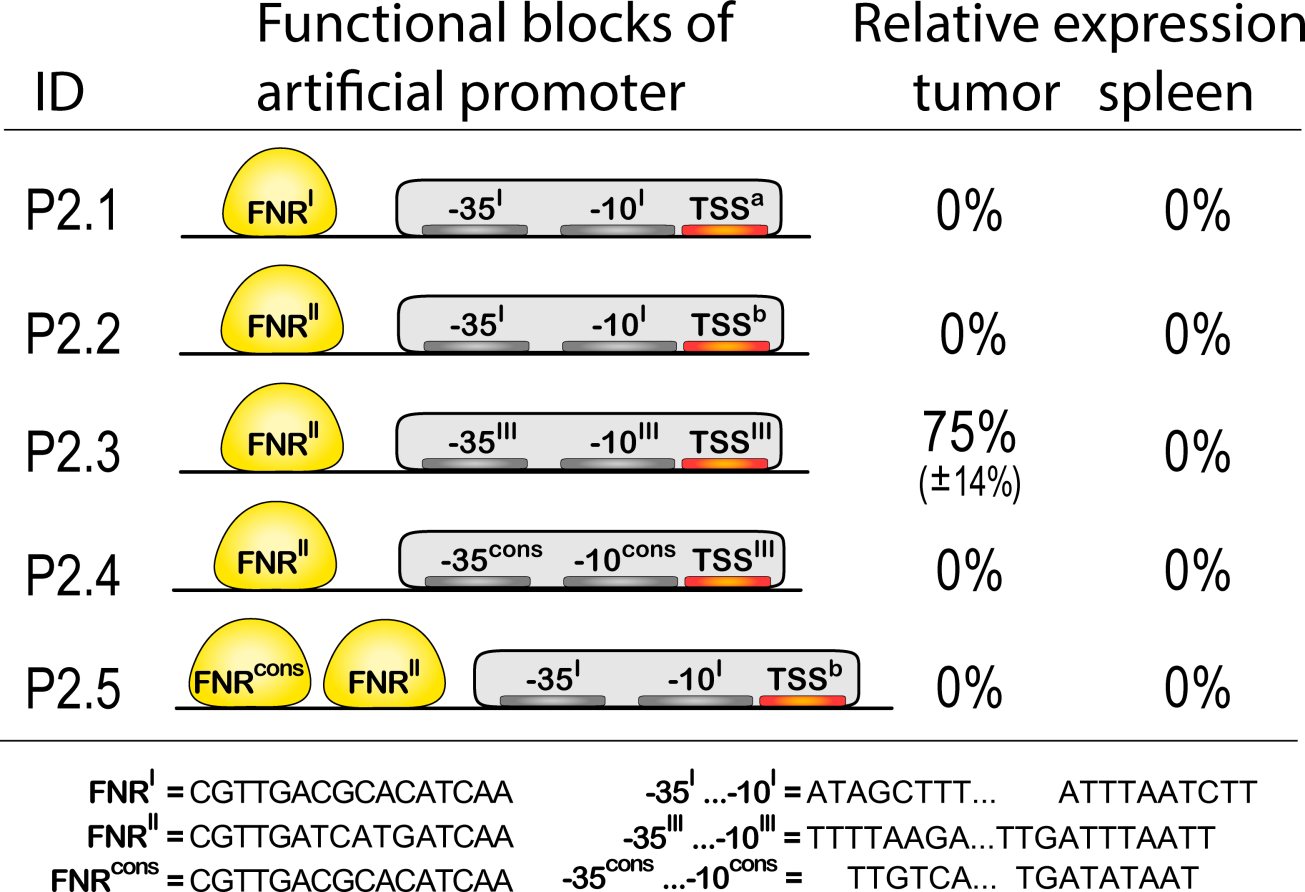
Schematic representation of artificial promoters and their expression in tumor environment. Promoters were compiled by introduction of the respective elements taken from promoters P0.212, P1.6, P0.134 as well as consensus sequences into the template sequence. Additionally, regions around TSS were enriched for A/T to facilitate DNA melting. Expression values are relative to the expression of P0.212_1. Given are mean±SD. Only promoter P2.3 showed expression in tumor. No expression in tumor or spleen could be observed for the other promoters. Nucleotide sequences are presented in the S3 Fig. Foot note: **I, II, III**—corresponding sequences were taken from promoters P0.212, P1.6, P0.134 respectively. **cons**—consensus sequences for the corresponding elements. **a, b**—modification of loci around putative start of transcription to facilitate DNA melting by random substitution of "C/G"s by "A/T"s.

Five promoters were constructed by implanting the FNR motif together with basal promoter elements into the template. We denote these as round 2 promoters "P2." (schematically shown in Fig 5). Basal promoters were constructed from short motifs representing -35 and -10 elements and a region of transcription initiation taken from P0.212, P0.134 or P1.6. One promoter was compiled using consensus sequences for the TATA box and *Inr* element (P2.4) and another one was complemented by an additional FNR consensus sequence (P2.5).

It is known that the region of transcription initiation is characterized by a low melting temperature that is achieved by a high AT content [24]. As was established above, tumor specific promoters indeed display higher AT content and contain many AT-rich regions. However, the template we selected exhibits a GC content of 0.55 and particularly a motif "GGTGGG" around the prospected start of transcription. We therefore randomly changed several nucleotides to A and in one case introduced a motif "AATAAAC" taken from the promoter P0.134 (Fig 5, sequences are given in S3 Fig). All fragments underwent the same cloning and testing procedure as before.

Results of the expression analysis are shown in Fig 5. All fragments that were constructed from basal promoter elements taken from the promoter P0.212 or from consensus sequences could not initiate any transcription. Such functional deficiency under all tested conditions (see also the next section) can only be explained by lack of functionality of basal promoters. The obvious explanation of this is a low prediction accuracy of the bioinformatic methods and the insufficient knowledge on basal promoter elements. The only promoter which showed transcriptional activity was P2.3, that was combined using elements from promoters P1.6 and P0.134. The DNA sequence of this promoter is AGACCAATGG ACATCCACGG CGATTATTAC GTTGATCATG ATCAAGCAGT TTTAAGACTA TACCAACTTG ATTTAATTCT TGTAATAAAC GAATGCC. Expression under control of this promoter is highly restricted to the tumor tissue. Absolute level of expression is approx. 75% compared to promoter P0.212 and 86% compared to P0.134. This demonstrates that the elements identified in the previous stage are necessary and sufficient for the specific transcriptional response in the tumor microenvironment. It opens the possibility for further development of specific promoters with highly individual expression profiles.

### *In vitro* experiments

**Fig 6:**
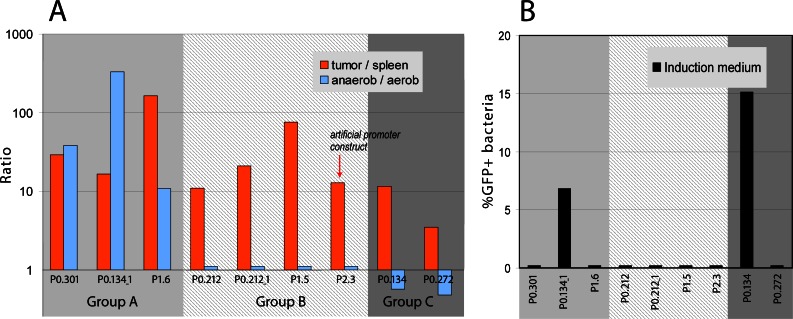
Activation of bacterial tumor-specific promoters under various conditions. **(A)** Expression ratios of tumor specific promoters in anaerobic and aerobic environments. For comparison, ratios for tumor and spleen are given. Three groups can be identified: group A–promoters that have similar expression (tumor—high; spleen—low; anaerobic -high; aerobic -low); group B–promoters that lost expression under anaerobic conditions and group C–promoters showing higher expression under aerobic conditions compared to anaerobic. Data were acquired 4 hrs after initiation of the cultures. (**B**) Promoter activation under acidic induction medium conditions. Only promoter P0.134 and its fragment P0.134_1 show expression. Promoter grouping is the same as in Fig 6A. Data were acquired 3 hrs after initiation of the cultures. The experiments were carried out twice under similar conditions. Results were essentially the same.

After tumor colonization, the bacteria are believed to reside in areas of low oxygen supply. To test the hypothesis that tumor specific promoters might be regulated exclusively by hypoxic conditions, we tested 14 selected constructs *in vitro* under aerobic and anaerobic culture conditions. We extended such tests also by using acidic induction medium as tumors might also present a microenvironment of low pH. Results were compared with the established *in vivo* situation in tumor and spleen and are shown in Fig 6A. Five promoters, namely P1.2, P2.1, P2.2, P2.4, P2.5, did not show any expression either under aerobic or anaerobic *in vitro* conditions and are not shown.

All other promoters could be divided into three functional groups. The first group of promoters showed high expression under anaerobic conditions and in tumors and low under aerobic conditions and in spleen (Fig 6A group A). In the second group, expression levels were similar under both *in vitro* conditions, but still strong differential expression was observed in tumors compared to spleen (group B). The third set of promoters showed even an increased level of expression under aerobic *in vitro* conditions. *In vivo* expression of such promoters was still restricted to the cancerous tissue (Fig 6A group C). This is somewhat surprising, since in the spleen aerobic conditions should be dominating.

We also tested all promoters for activation in induction and minimal medium which might mimic the low nutrient supply and the low pH encountered in a tumor. Only promoter P0.134 and its fragment P0.134_1 were activated when cultivated in induction and minimal medium (Fig 6B, data for minimal medium are similar and not shown).

These experiments indicate that many of our promoters specifically respond to additional, presently unknown, factors encountered in tumor environments. Interestingly, the artificial promoter P2.3 that is fully functional in tumors but not in spleen is not sensitive to low oxygen conditions nor is it responding to induction medium.

### Histological analysis

**Fig 7:**
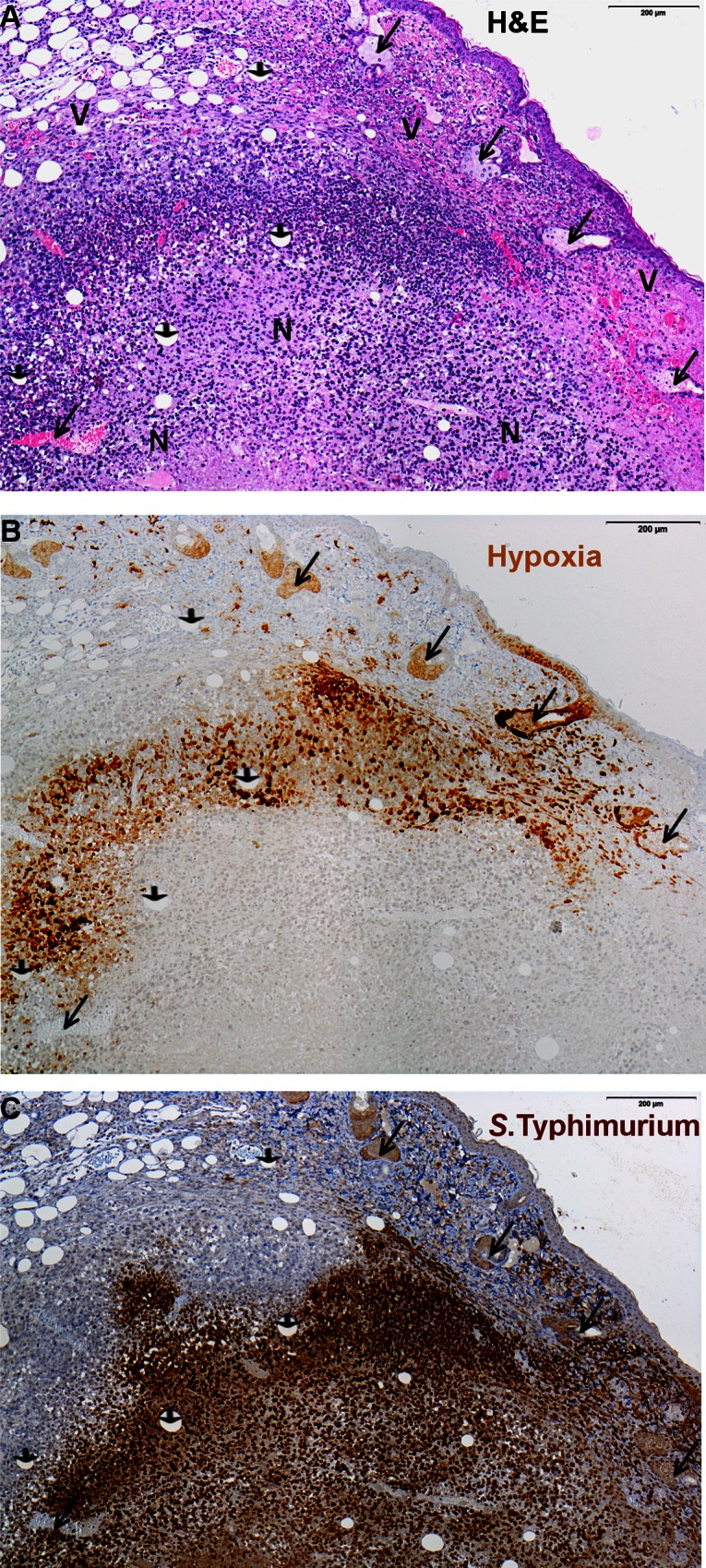
Localization of *Salmonella* expressing GFP under control of P0.212_1 within various regions of solid murine tumors. Consecutive tumor sections are shown. (**A**) Hematoxylin and eosin (H&E) staining showing infiltration of live immune cells (closed purple nuclei) between viable (V) and necrotic (N) tumor zones. (**B**) Immunochemical detection of hypoxic tissue (light brown staining) by a rabbit-anti-pimonidazole antibody and (**C**) *S*. Typhimurium strain SL7207 cells (dark brown) by a rabbit-anti-salmonella antibody. Here, arrow heads indicate vessles, long arrows indicate sebaceous glands.

These heterogeneous results prompted us to investigate in which tumor region the *Salmonella* are precisely localized. Therefore, colonized tumor tissue was analyzed by histology. An accumulation of immune cells mainly consisting of neutrophils was visualized by hematoxylin and eosin (H&E) staining between necrotic and viable tumor zones (Fig 7A). Partially overlapping with this zone, a large hypoxic region could be detected with a similar shape as the leukocyte zone (Fig 7B). Additionally, this hypoxic region bordered on the necrotic tumor zone where no viable cells were present and which is most likely anoxic (Fig 7A and 7B). *Salmonella* apparently colonize the hypoxic region of the tumor as well as the anoxic necrotic zone (Fig 7C). Thus, the bacteria colonize a very heterogeneous environment which is consistent with our finding that the promoters are activated by heterogeneous factors, only some of which are evident.

The presented results indicate that we have identified critical elements of tumor specific promoters. We also show that apparently other DNA features are present in particular TSP promoters that render some of them responsive to hypoxic or induction media conditions. Our data also suggest that there are features, probably distributed along the promoter sequences that quantitatively influence the level of expression. The artificial promoter that lacks these features responds exclusively to the tumor microenvironment that was proved in all experiments. Understanding these features may shed light on attributes of the tumor microenvironment that may distinguish solid tumors from other tissues.

## Discussion

### Probable mechanism of tumor specific activation

According to the bioinformatic and experimental results we may speculate on how tumor specific activation is achieved. In normal tissues, a level of the active dimerized form of FNR protein is low and the promoter receives no activation signals. To avoid leakage, the basal promoter should be inefficient enough such that it is not able to initiate transcription by itself, since no repressor element is found in the promoters. The "extent of inefficiency" is presumably very vague and cannot be defined as a number of mismatches from consensus *Inr* or TATA-box sequences. Once, a boost signal from FNR is received, for example, under anaerobic conditions, some promoters already show pronounced transcription (Fig 6A group A). For other promoters, activation only by FNR is still not sufficient (Fig 6A group B). However, they are transcriptionally active in the tumor microenvironment where additional factors play a role and the overall signal is sufficient enough to initiate transcription. The mechanism of additional factors also agrees with our data on mutation of "insignificant" nucleotides (P1.6 and P1.7, Fig 3) that led to significant changes in transcriptional activity. One of the reasons for this could be a change in overall physico-chemical properties of a DNA stretch that is shown to significantly influence transcription [25, 26]. But could such factors initiate transcription by themselves? The absence of expression of promoter P1.2 (FNR knock out) in tumor, spleen and under an- and aerobic conditions shows that FNR is a compulsory prerequisite for transcription. Altogether: DNA tertiary structure and nucleotide context within and around the basal promoter serve as a trigger under the special conditions realized solely in tumors. Further we discuss some of the factors in more detail.

### Efficient binding of FNR

In the absence of oxygen FNR protein forms dimers and only this active state promotes gene expression [21]. Modifications of nucleotides in the middle of the FNR binding motif led to an increased level of expression (Fig 3, P1.6). The modified fully symmetrical FNR motif is supposed to bind the FNR dimer more efficiently [21] and this might explain the intensified transcription.

### DNA remodeling

A single nucleotide deletion in TGIF motif (P1.5, Fig 3) led to the reduction of expression by 25%. TGIF is a eukaryotic transcription factor and most probably is not relevant in this context. But the motif itself "CTTTGTCAGAA" contains a conserved triplet "TGT" which is known to significantly bend DNA [27]. A specifically bended DNA of a promoter region can initiate transcription more effectively by more efficient binding the CAP protein [27]. Besides TGT, there are other regions not covered by the identified motifs that contribute to the rate of transcription, but not to the specificity. This can be concluded from the mutations of "insignificant" nucleotides in promoter P1.7 which led to a significant reduction in the level of expression.

### Weak basal promoter

TSP promoters exhibit relatively low promoter recognition score of 82.7±5.5 (SEM) as identified by the BROM program [13]. So for example, core promoter elements of the well studied P0.212 are TAGCTT (-35) and TTTAAT (-10) and appeared to be not optimal compared to the known consensus sequences TTGTCA and TATAAT. To compare: promoter recognition score for the *Salmonella* housekeeping genes is 95.3±3.4 (genes: *aroC*, *dnaN*, *hemD*, *hisD*, *purE*, *sucA* and *thrA* [28], for NP promoters is 81.1±3.7 and for RP promoters is 78.2±7.9.

From another side, the overall score revealed by the kernel method [11] of the TSP promoters is significantly higher compared to NP or RP promoters (see results section). The latter program additionally accounts for nucleotides between and around of -35 and -10 elements. Therefore, we may suggest that the specificity of expression of TSP promoters is achieved by very fine tuning of the basal promoter (in-)efficacy. This also explains why a quite frequent combination of FNR and normal promoter do not provide required tumor specificity.

The deviation from the well known assumption that promoters should mainly respond to anaerobic conditions, known as Warburg effect, is interesting. Promoters of group A in Fig 6 respond to anaerobic conditions as predicted, promoters in groups B and C do not, but all promoters respond to tumor conditions. It demonstrates that in the tumor microenvironment other conditions exist which make promoters active. Osmolaritiy and pH are known to be distinct between normal tissue and neoplasias and could be a reason for the activation via specific DNA remodeling. In addition, the insufficient nutrient supply as mimicked by minimal medium might trigger some regulatory mechanisms. We could also show that tumors colonized by bacteria strongly attract neutrophilic granulocytes [29]. Signals from such cells might also induce transcription via anti-microbial peptides or other secreted molecules. Thus, molecular definition of such additional transcriptional inducers will lead to a more complete picture of tumor microenvironment.

Obviously, promoters of *Salmonella* are not evolutionary selected for the microenvironment of a solid tumor. Rather, the tumor mimics natural habitats of the bacteria. Hypoxia and anoxia, as can be found in the central necrotic or its neighboring regions, was a first apparent suggestion by us and others. Such conditions might not prevail in systemic organs but are most likely excessive in the large intestine. This idea was only partly confirmed. Apparently, the tumor microenvironment represents a highly complex environment for which a natural equivalent cannot be envisioned yet. It will be important to unravel such conditions further as it may provide new targets for therapy by bacteria or other means.

## Material and Methods

### Ethics statement

Procedures involving animals and their care were fully in compliance with the German Animal Welfare Act (Tierschutzgesetz, 1998) and with the permission number 33.9.42502-04-050/09 of LAVES (Niedersaechsisches Landesamt fur Verbraucherschutz und Lebensmittelsicherheit).

### Construction of insert fragments

To construct plasmids that contain fragments of the original library inserts [30], oligonucleotides of the desired sequence were either directly ordered (Eurofins MWG Operon, Germany) and cloned into the vector (pMW82), or for longer sequences, primers were designed accordingly to amplify the fragment from the original plasmid. SL7207 was transformed with plasmids containing the amplification products and plasmid DNA was sequenced to confirm correct sequence of the amplification products.

### Animal experiments

Eight weeks old female BALB/c mice were purchased from Janvier (France) and subcutaneously injected with 5x10^5^ CT26 colon carcinoma cells (ATCC CRL-2638). When tumors reached volumes of approximately 200 mm^3^, mice were infected intravenously with 5x10^6^ bacteria (*Salmonella* Typhimurium strain SL7207) in 100 μl PBS. One, three, and five days after infection, mice were sacrificed by exposure to CO_2_, respective tissues were removed and homogenized in 2 ml PBS. The homogenates were diluted 1:10 (spleen, liver) or 1:100 (tumors) in 0.1% TritonX-100/PBS containing 2 mM EDTA, filtered through a 30 μm CellTrics filter (Partec, Germany) and sorted. Samples were analyzed via two color flow cytometry on a FACSAria or LSRII, respectively (Becton Dickinson, USA) and plated on LB plates containing 50 μg/ml ampicillin to allow normalization. No plasmid loss was confirmed via plating on ampicillin. The two color flow cytometry is a method that allows to discriminate GFP expressing bacteria from autofluorescent cellular debris since GFP expressing *Salmonella* have a substantially lower orange/green emission ratio [31]. Additionally, forward and side scatter were used to discriminate *Salmonella* from larger particles by setting an appropriate scatter gate. For more detailed information see [8]. For histological analyses, mice received 1d p.i. 1.5 mg of the hypoxia marker pimonidazole hydrochloride (Hydroxyprobe, Inc) dissolved in 100 μl saline. The tumors were harvested 45 min after administration, fixed in 4% neutrally buffered formaldehyde for 24 to 48 hours, embedded in paraffin and consecutive 3 μm sections were stained with the affinity purified rabbit-anti-pimonidazole antibody (PAb2627AP 0.5mg/ml IgG), rabbit-anti-salmonella sp. antibody or hematoxylin-eosin. Sections were analyzed by light microscopy with an Olympus BX51 microscope and cellSens software.

### Bacterial growth under aerobic and anaerobic conditions

Respective bacterial strains were streaked out from glycerol stocks onto LB agar plates containing the appropriate antibiotics. After overnight growth at 37°C, the cultures were used to inoculate (i) 4 ml LB medium with antibiotics and grown at 37°C overnight with shaking at 180 rpm or (ii) 15 ml of induction medium (IM) and minimal medium (MM). Both are M9 medium based [32] without CaCl_2,_ supplemented with the appropriate antibiotics, 100 μM MgSO_4_, 40 μg/ml histidine, 40 μg/ml phenylalanine, 40 μg/ml tryptophane, 40 μg/ml tyrosine, 10 μg/ml para-aminobenzoic acid, 10 μg/ml 2,3-dihydroxybenzoate and 0.2% glucose. The salt concentration was decreased to 0.05% NaCl and the pH was adjusted to 5.5 (IM) or to 7.4 (MM). From the 4 ml liquid cultures (i) 200 μl were used to start two new cultures of 20 ml LB medium each. One was grown under aerobic and the other under anaerobic conditions. Before 1:100 inoculation of the 15 ml liquid cultures for condition (ii), the cultured bacterial cells were washed twice in PBS and adjusted to OD_600_ of 1.0. Cultures were analyzed at different time points by flow cytometry and parallel by OD_600_ measurements or plating to allow normalization. Data presented were derived from 3 hrs (minimal medium) or 4 hrs (aerobic/anaerobic) cultures, respectively.

### Bioinformatics analysis

Datasets of promoter sequences were compiled on the basis of our previous research. According to that, tumor specific promoters (TSP) are 13 promoters from class 1, 115 negative promoters (NP) are from class 5 and lowTSPs are 12 promoters from class 2 [8]. LowTSP promoters show lower expression in tumors than TSP and may additionally have some low but non-zero expression in spleen. A random promoter dataset (RP) was compiled by splitting randomly the entire *Salmonella* genome into fragments following the same length distribution as in the TSP set, resulting in 7682 sequences. Negative promoters (NP) are DNA fragments from the *Salmonella* genome that are proved not to initiate any transcription either in tumors or spleen [8].

Promoter nomenclature will be as follows. Promoters from [8] will be denoted as "P0." (round 0) followed by a number that corresponds to the number used in [8]. Fragments of P0. promoters will be supplemented with a consecutive number (for example, P0.212_1). Promoters in knockout experiments will be denoted as P1., artificial promoters as P2. both followed by a consecutive number.

Parameters of the methods for recognition of basal promoters, regulatory motifs and other elements were selected such that they maximize discrimination between tumor specific promoters and negative promoters. As boundary condition, it was set that at least 75% (10 out of 13) of TSPs must have a minimum of one recognized element and at most 50% of NPs may contain such an element. We will denote a portion of TSPs that have an element as *ElementName*^*TSP*^ and for negative promoters as *ElementName*^*NP*^. Recognition was performed for a range of values for each parameter required by a method and those values that maximize the ratio *ElementName*^*TSP*^*/ElementName*^*NP*^ were selected as optimal, provided that the boundary conditions are met (i.e. *ElementName*^*TSP*^ ≥0.75 and *ElementName*^*NP*^≤0.50). The higher the ratio the more specific is an element to the promoters.

This principle was applied for recognition of basal promoters using Kernel [11] and HMM [12] methods, for the identification of DNA binding motifs using position weight matrixes (PWMs) and for the evaluation of AT-rich regions. AT-rich regions were defined as 100bp regions with an overall A+T content over 0.6. When searching for the repeat AAAAAAAA (we denote A_8_), one mismatch is allowed. To identify exact positions of TATA-box and *Inr* element program BROM [13] was applied, which is developed by the same authors as the sequence alignment kernel [11]. Due to limitations of the program it could not be applied to batch processing, but only to single promoters.

## Supporting Information

S1 Fig Motifs identified in the set of tumor specific promoters. Values are normalized numbers of promoters in a set containing at least one motif. P–values are calculated as a binomial probability to observe the actual number of promoters with a motif in the TSP set compared to RP set.
(DOC)

S2 Fig DNA sequence and nucleotide substitutions introduced into the minimal promoter and results of expression analysis. DNA motifs for FNR are shown in yellow, NagC in light brown, TGIF in green, TATA-box and Inr element in grey. Mutated nucleotides are shown in red.
(DOC)

S3 Fig DNA sequence of artificial promoter constructs. DNA motifs for FNR are shown in yellow, -35 and -10 elements are in grey. Nucleotides introduced into the template sequence are in capital and marked red.
(DOC)

S1 Table Genes associated with tumor specific promoters. (DOC)

S2 Table Combinatorial modules found in tumor specific promoters. Each module consists of two or more elements indicated by "+". P–values are calculated as a binomial probability to observe the actual number of promoters with a module in the TSP set compared to RP set.
(DOC)

S1 Text DNA sequences of the tumor specific promoters. (DOC)
