# Supplementary material for: Composing a Tumor Specific Bacterial Promoter
Source: PLoS One. 2016 May 12;11(5):e0155338. doi: 10.1371/journal.pone.0155338 (PMC4865170; doi:10.1371/journal.pone.0155338)
Supplement: S1 Fig — Values are normalized numbers of promoters in a set containing at least one motif. P–values are calculated as a binomial probability to observe the actual number of promoters with a motif in the TSP set compared to RP set. (DOC) [file pone.0155338.s001.doc]

**Fig S1.** **Motifs identified in the set of tumor specific promoters.** Values are normalized numbers of promoters in a set containing at least one motif. *P*-values are calculated as a binomial probability to observe the actual number of promoters with a motif in the TSP set compared to RP set.

| Program or library | Motif name | Motif logo | Frequency in TSP | Frequency in NP | *p*-value |
| --- | --- | --- | --- | --- | --- |
| E.coli PWM library | NagC | 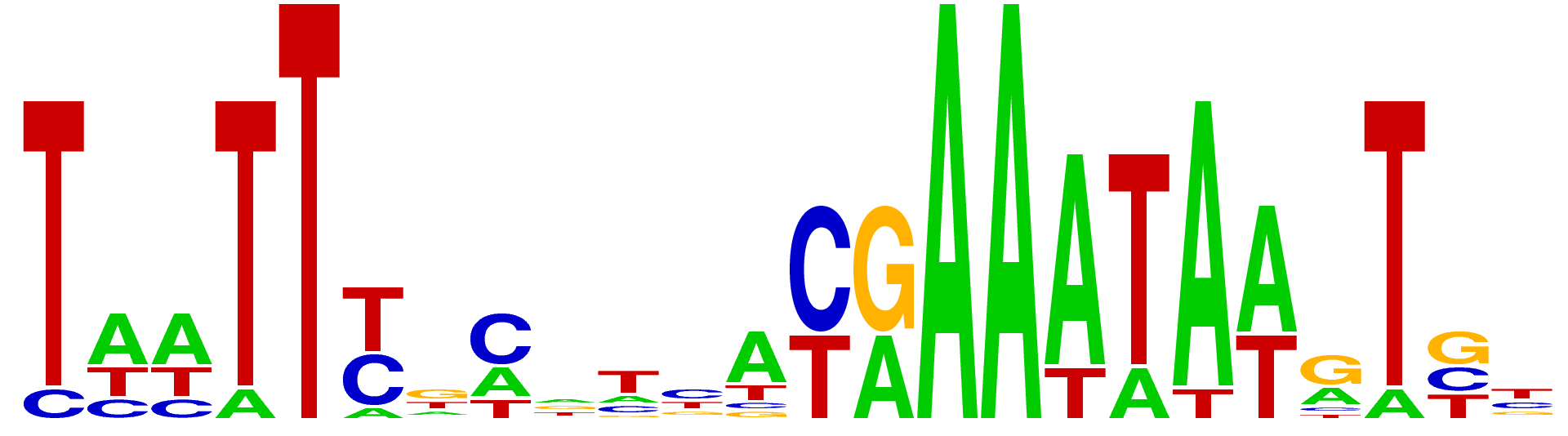 | 0.92 | 0.28 | 2.2*10^-6^ |
|  | RscAB | 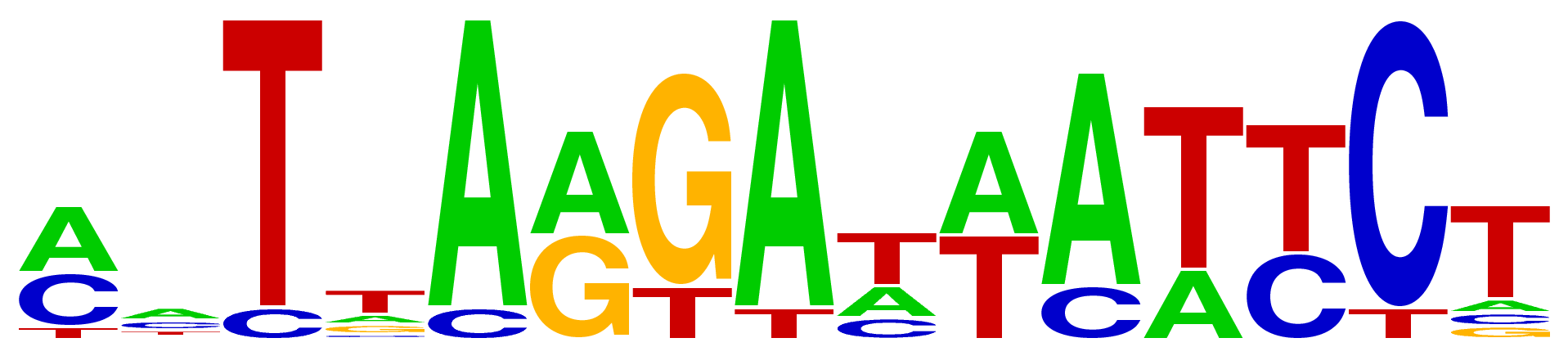 | 0.85 | 0.40 | 1.4*10^-4^ |
|  | DnaA | 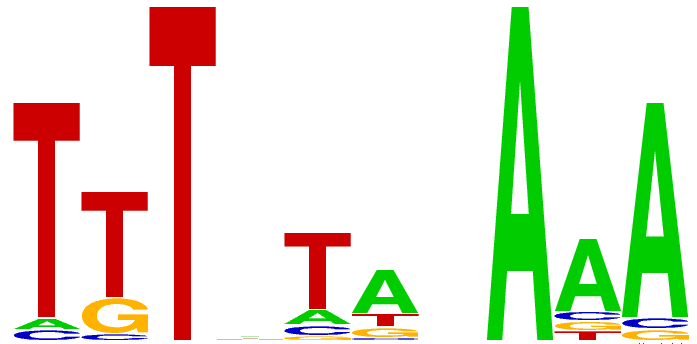 | 0.77 | 0.36 | 4.6*10^-04^ |
|  | FNR | 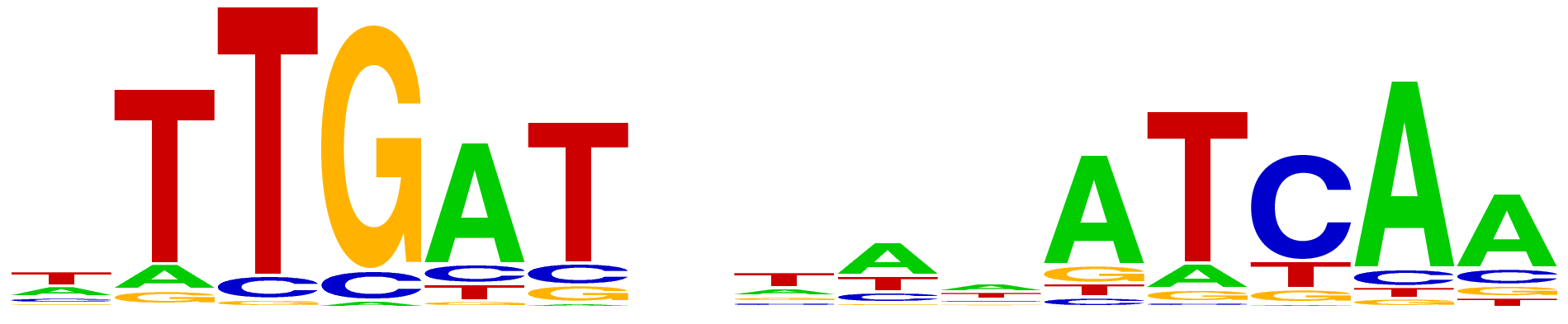 | 0.85 | 0.32 | 1.1*10^-5^ |
| Eukaryotic PWM library | MEF2 | 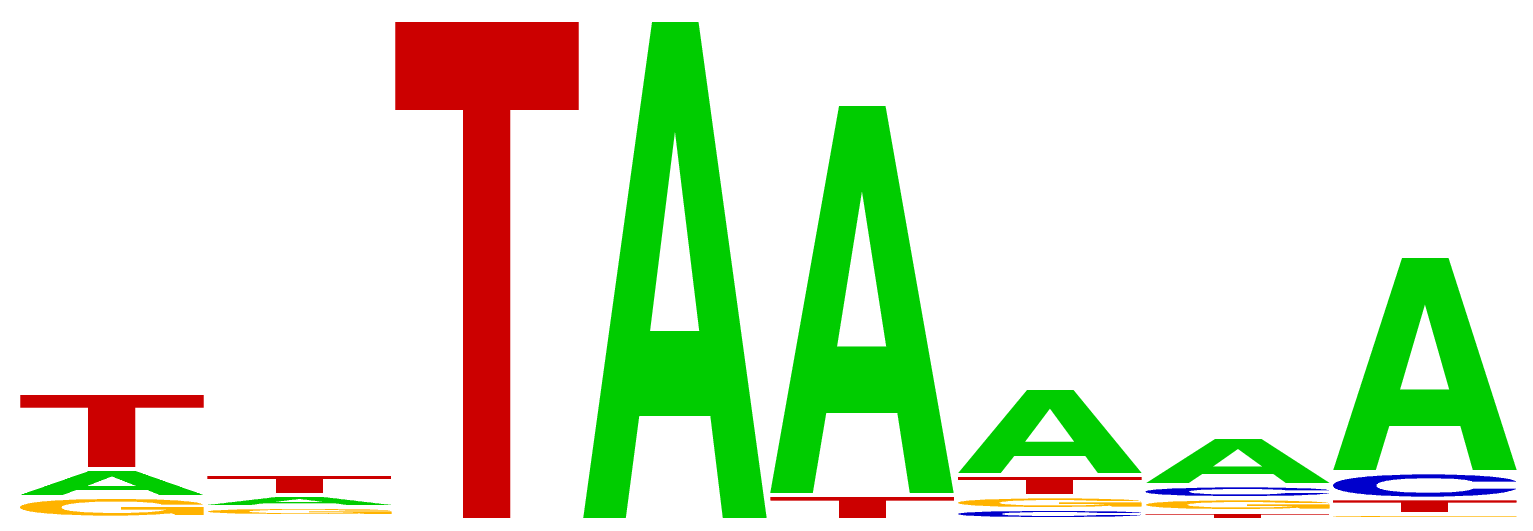 | 0.85 | 0.34 | 2.1*10^-5^ |
|  | TGIF | 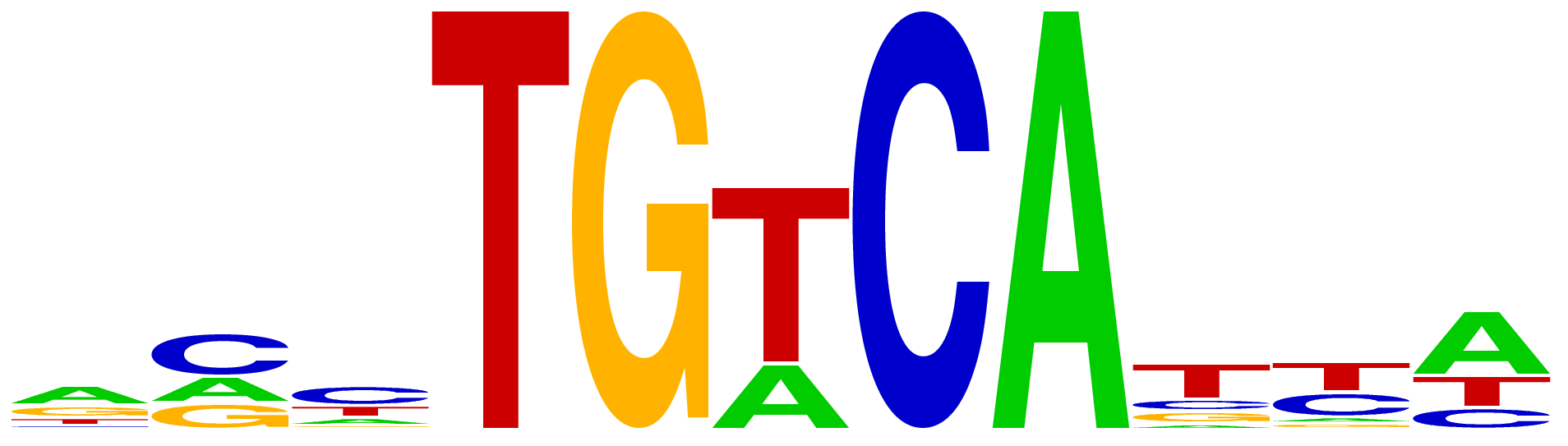 | 0.77 | 0.28 | 3.6*10^-5^ |
|  | TEF | 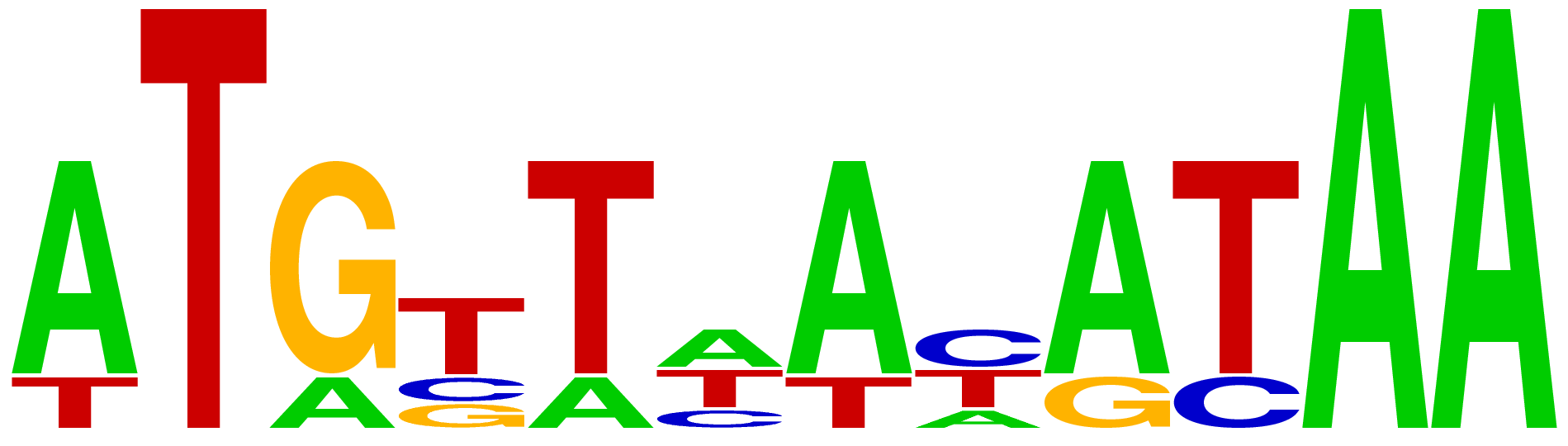 | 0.77 | 0.17 | 1.9*10^-7^ |
|  | SOX9 | 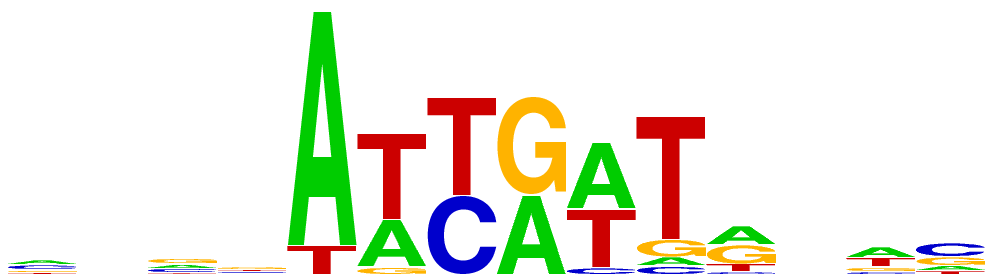 | 0.77 | 0.47 | 6.3*10^-3^ |
|  | HNF1 | 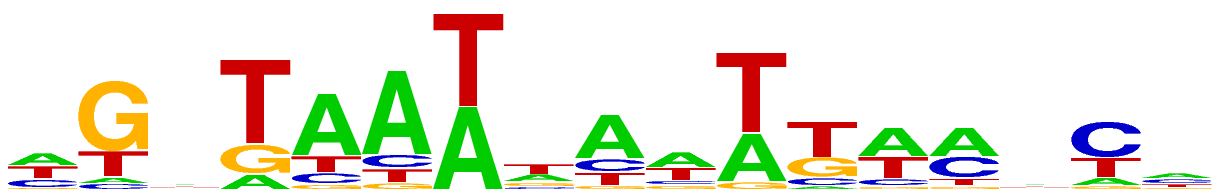 | 0.77 | 0.50 | 1.1*10^-2^ |
|  | BRCZ4 | 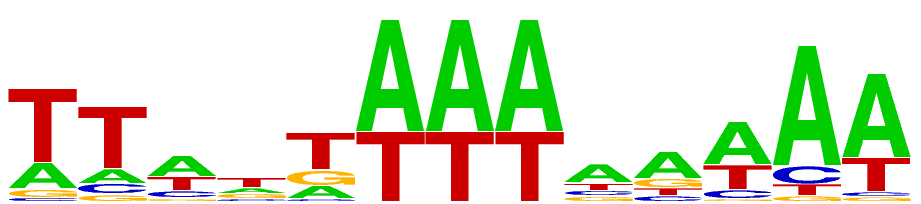 | 0.77 | 0.43 | 2.7*10^-3^ |
| MEME | Meme1 | 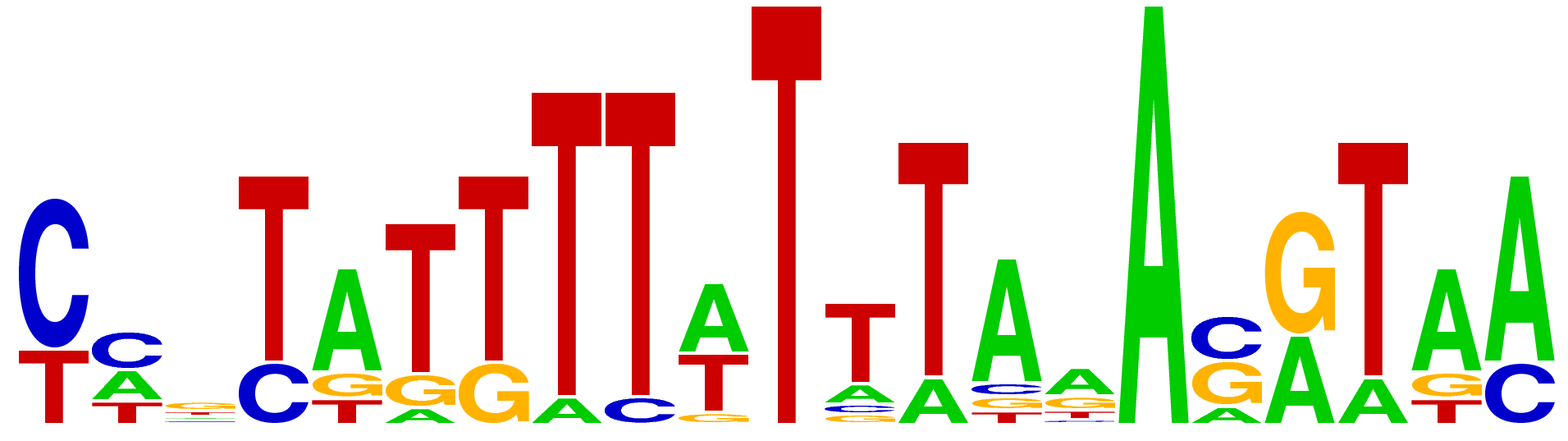 | 0.85 | 0.06 | 2.7*10^-14^ |
|  | Meme2 | 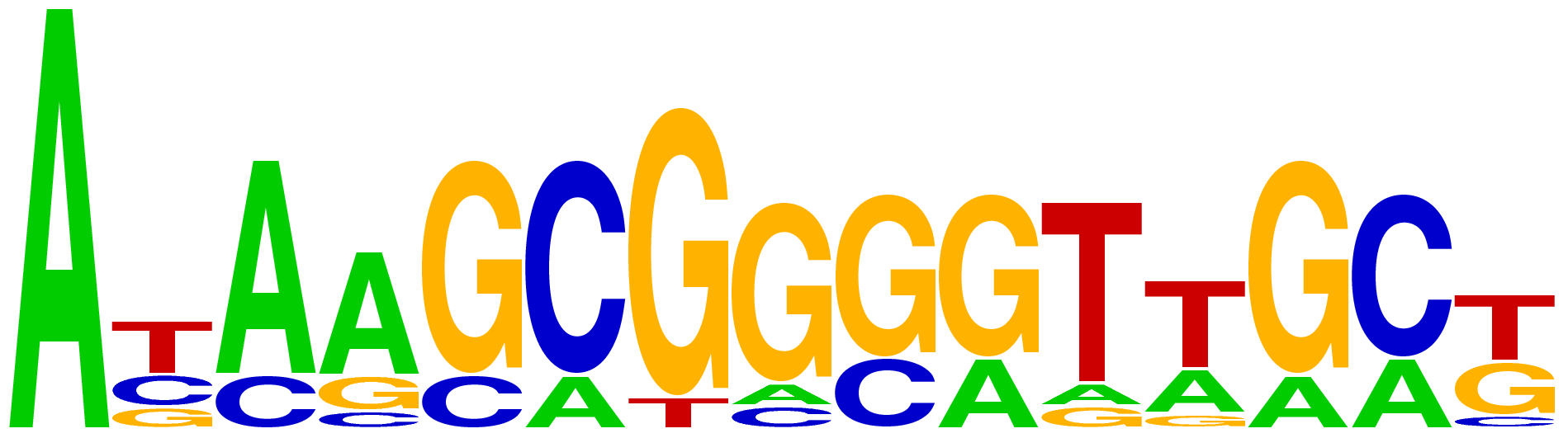 | 0.77 | 0.18 | 3.5*10^-7^ |
|  | Meme4 | 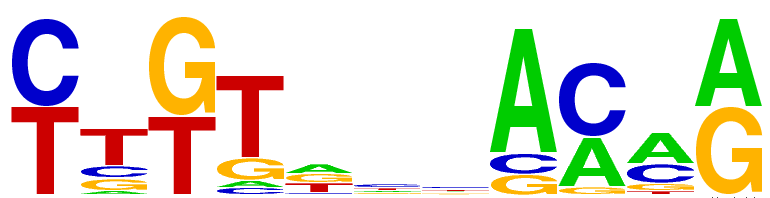 | 0.77 | 0.34 | 2.6*10^-4^ |
|  | Meme5 | 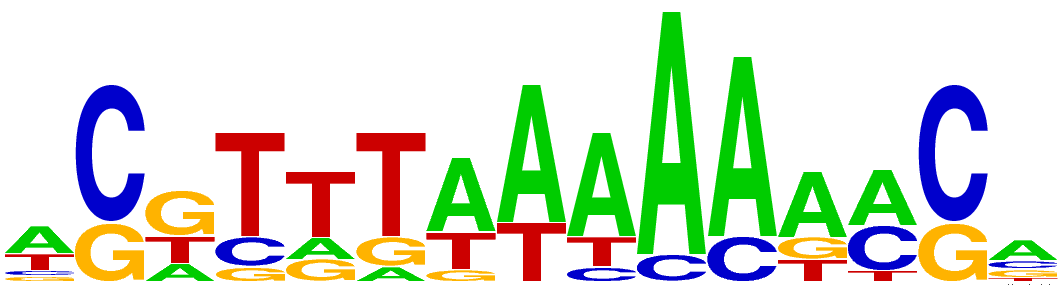 | 0.77 | 0.15 | 5.0*10^-8^ |
|  | Meme6 | 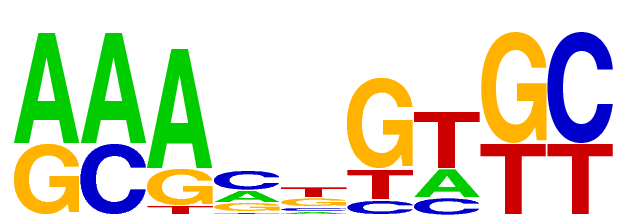 | 0.92 | 0.26 | 9.4*10^-7^ |
|  | Meme9 | 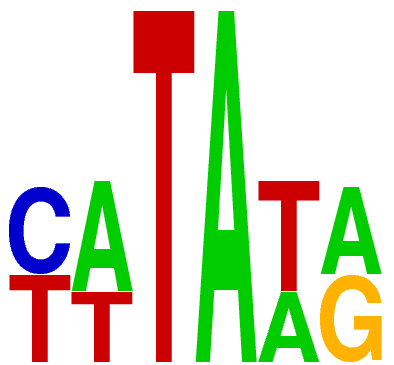 | 0.85 | 0.35 | 3.0*10^-5^ |
| MDScan | MDScan2 | 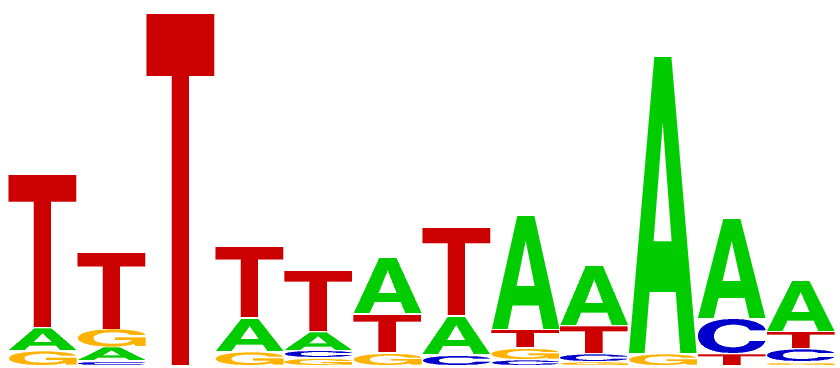 | 0.85 | 0.14 | 6.4*10^-10^ |
|  | MDScan3 | 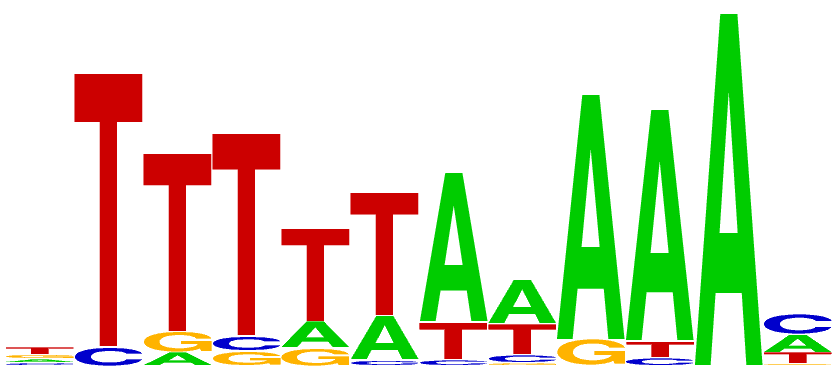 | 0.77 | 0.08 | 5.8*10^-11^ |
|  | MDScan5 | 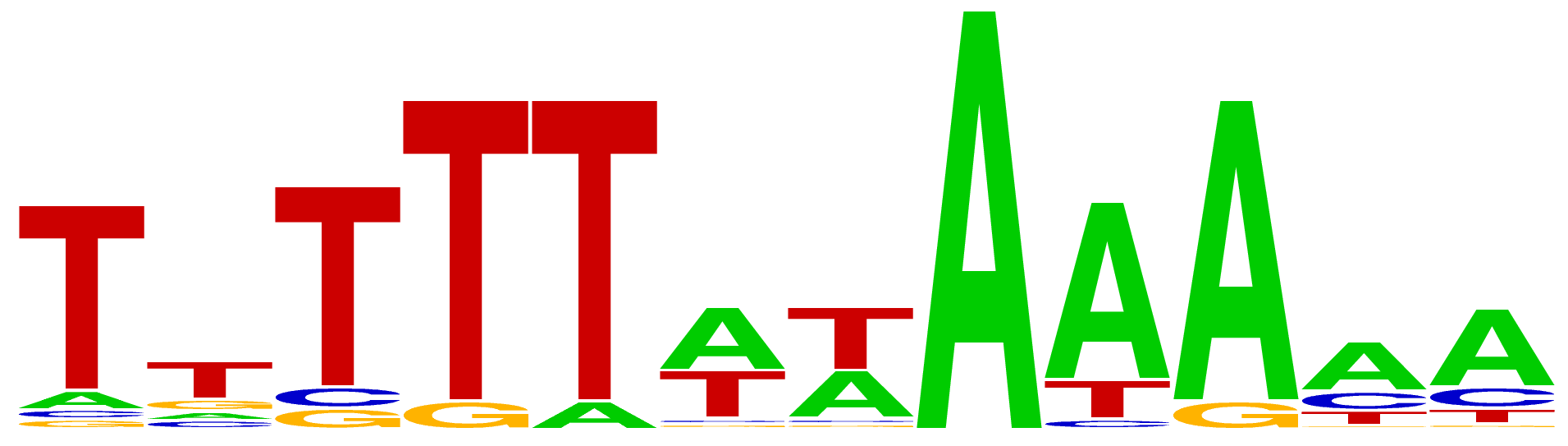 | 0.85 | 0.11 | 3.7*10^-11^ |
|  | MDScan8 | 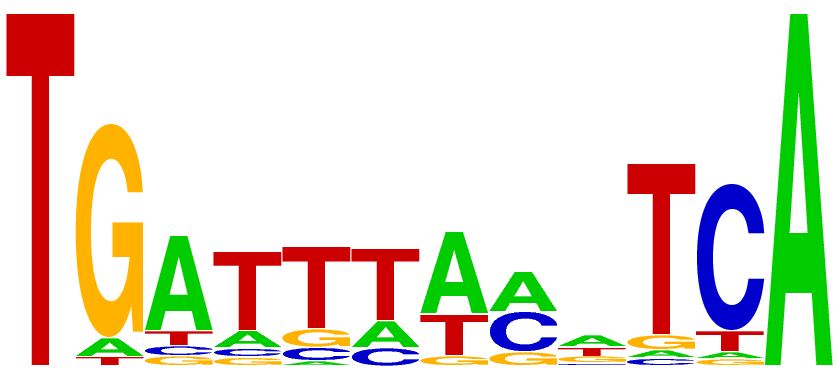 | 0.92 | 0.32 | 1.1*10^-5^ |
|  | MDScan10 | 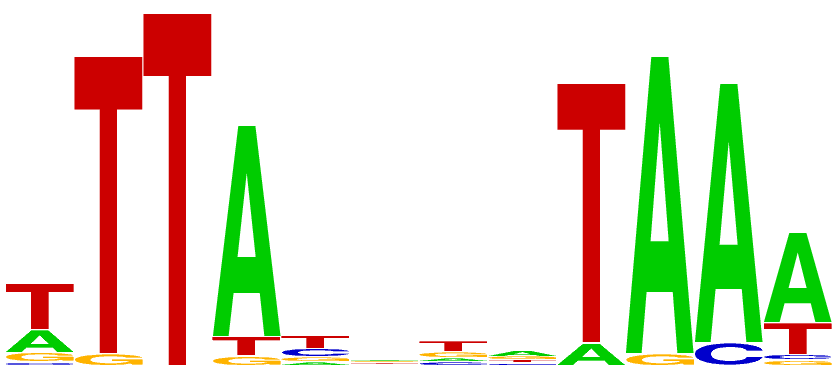 | 0.77 | 0.17 | 1.9*10^-7^ |
| DME | DME1 | 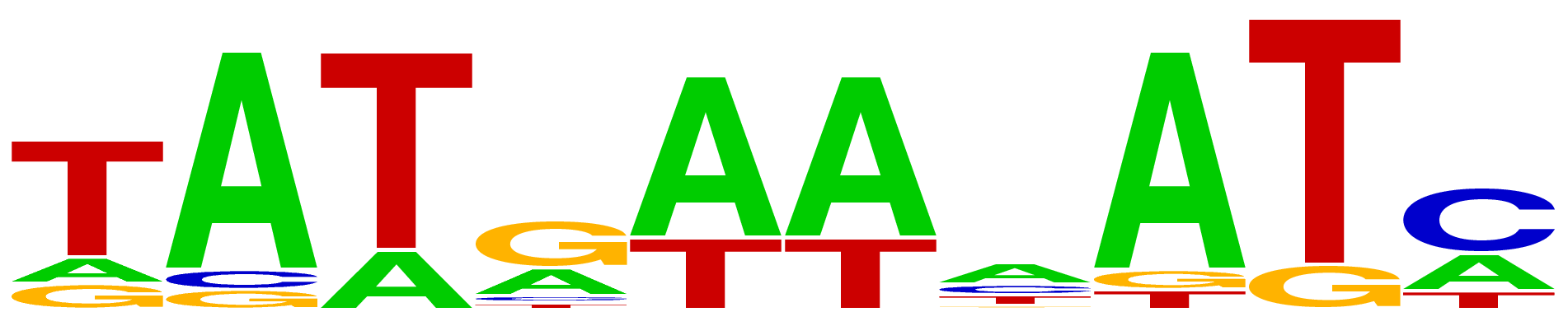 | 0.85 | 0.15 | 1.5*10^-9^ |
|  | DME 2 | 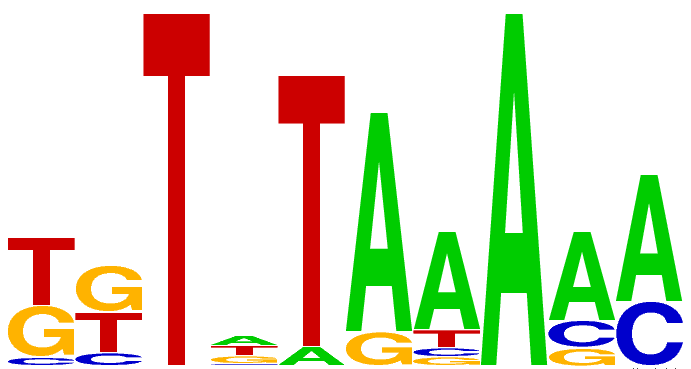 | 0.85 | 0.16 | 3.1*10^-9^ |
| CMF | CMF1 | 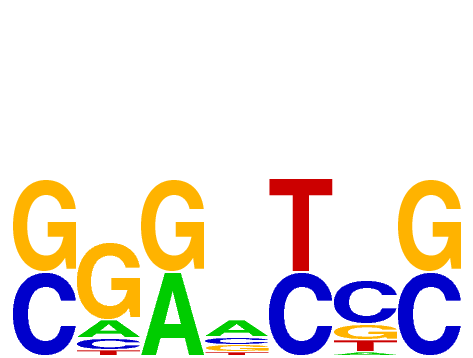 | 0.92 | 0.35 | 3.0*10^-5^ |
|  | CMF11 | 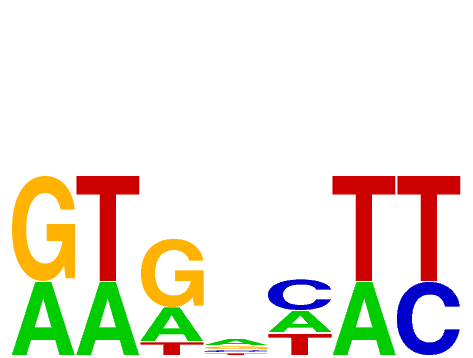 | 0.75 | 0.19 | 9.9*10^-6^ |
