# Supplementary material for: Composing a Tumor Specific Bacterial Promoter
Source: PLoS One. 2016 May 12;11(5):e0155338. doi: 10.1371/journal.pone.0155338 (PMC4865170; doi:10.1371/journal.pone.0155338)
Supplement: S2 Fig — DNA motifs for FNR are shown in yellow, NagC in light brown, TGIF in green, TATA-box and Inr element in grey. Mutated nucleotides are shown in red. (DOC) [file pone.0155338.s002.doc]

**Fig S2.** **DNA sequence and nucleotide substitutions introduced into the minimal promoter and results of expression analysis.** DNA motifs for FNR are shown in yellow, NagC in light brown, TGIF in green, TATA-box and *Inr* element in grey. Mutated nucleotides are shown in red.

| Seq Id | Changes in DNA sequence | Expression in | |
| --- | --- | --- | --- |
|  |  | tumor | spleen |
|  | Original promoter:  taacgcct**CTTTGTCAGA**acctctccattc**GTTGAcgcacATCAA**ga**tagctt**tcattcgaaagtaa**TtTAATctttatATGaaataagag**aggccgttt | 100% | 0% |
| P1.1 | mutation of NagC**:**  taacgcct**CTTTGTCAGA**acctctccattc**GTTGAcgcacATCAA**ga**tagctt**tcattcgaaagtaa**TtTAATctttatATGaaatGCgag**aggccgttt | 100% | 0% |
| P1.2 | mutation of FNR:  taacgcct**CTTTGTCAGA**acctctccattc**GCTGAcgcacATCAG**ga**tagctt**tcattcgaaagtaa**TtTAATctttatATGaaataagag**aggccgttt | 0% | 0% |
| P1.3 | mutation of both FNR and NagC**:**  taacgcct**CTTTGTCAGA**acctctccattc**GCTGAcgcacATCAG**ga**tagctt**tcattcgaaagtaa**TtTAATctttatATGaaatGCgag**aggccgttt | 0% | 0% |
| P1.4 | mutation of TATA-box:  taacgcct**CTTTGTCAGA**acctctccattc**GTTGAcgcacATCAA**ga**tagctt**tcattcgaaagtaa**TGTCAGctttatATGaaataagag**aggccgttt | 0% | 0% |
| P1.5 | deletion in TGIF**:**  taacgcct**CTTT–TCAGA**acctctccattc**GTTGAcgcacATCAA**ga**tagctt**tcattcgaaagtaa**TtTAATctttatATGaaataagag**aggccgttt | 75% | 0% |
| P1.6 | mutation of insignificant nucleotides in FNR and NagC**:**  taacgcct**CTTTGTCAGA**acctctccattc**GTTGATCATGATCAA**ga**tagctt**tcattcgaaagtaa**TtTAATctttatGATACataagag**aggccgttt | 160% | 0% |
| P1.7 | mutation of regions between motifs:  taagtcac**CTTTGTCAGA**acaacgctactc**GTTGAcgcacATCAA**ga**tagctt**tcactagtaagtaa**TtTAATctttatATGaaataagag**aggccgttt | 40% | 0% |
