# Supplementary material for: Composing a Tumor Specific Bacterial Promoter
Source: PLoS One. 2016 May 12;11(5):e0155338. doi: 10.1371/journal.pone.0155338 (PMC4865170; doi:10.1371/journal.pone.0155338)
Supplement: S3 Fig — DNA motifs for FNR are shown in yellow, -35 and -10 elements are in grey. Nucleotides introduced into the template sequence are in capital and marked red. (DOC) [file pone.0155338.s003.doc]

**Fig S3**. **DNA sequence of artificial promoter constructs.** DNA motifs for FNR are shown in yellow, -35 and -10 elements are in grey. Nucleotides introduced into the template sequence are in capital and marked red.

| Id | Changes in DNA sequence | Expression in | |
| --- | --- | --- | --- |
|  |  | tumor | spleen |
|  | template DNA sequence:  agaccaatggacatccacggcgattattacccaacctgatgccgcgcaggttgccacctataccaacgcgctcaacgtcttgtatggtgggaatgcc |  |  |
| P2.1 | agaccaatggacatccacggcgattatta**CGTTGACGCACATCAA**g**ATAGCTTT**cacctataccaa**ATTTAATCTT**gtcttgtat**AA**t**AA**gaatgcc | 0% | 0% |
| P2.2 | agaccaatggacatccacggcgattatta**CGTTGATCATGATCAA**g**ATAGCTTT**cacctataccaa**ATTTAATCTT**gtcttgtat**AAGTT**gaatgcc | 0% | 0% |
| P2.3 | agaccaatggacatccacggcgattatta**CGTTGATCATGATCAA**gcag**TTTTAAGA**ctataccaac**TTGATTTAATT**cttgt**AATAAAC**gaatgcc | 75% | 0% |
| P2.4 | agaccaatggacatccacggcgattatta**CGTTGATCATGATCAA**gcagg**TTGTCA**cctataccaacgcgct**TGaTATAAT**gtat**AAGTT**gaatgcc | 0% | 0% |
| P2.5 | aggcgaatttaagaaCGTTGACGCACATCAAga  aatggacatccacggcgattattaCGTTGACGCACATCAAgATAGCTTTcacctataccaaATTTAATCTTgtcttgtatAAGTTgaatgcc | 0% | 0% |
