# Supplementary material for: Composing a Tumor Specific Bacterial Promoter
Source: PLoS One. 2016 May 12;11(5):e0155338. doi: 10.1371/journal.pone.0155338 (PMC4865170; doi:10.1371/journal.pone.0155338)
Supplement: S1 Table — (DOC) [file pone.0155338.s004.doc]

**Table S1.** **Genes associated with tumor specific promoters.**

| Promoter | Gene name | Functional description |
| --- | --- | --- |
| P0.4 | *ydiH* | Putative cytoplasmic protein |
| P0.48 | *lpT* | n-glycerol-3-phosphate transporter |
| P0.92 | *csG* | Membrane protein; endoglucanase |
| P0.134 | *mltD* | Membrane-bound lytic murein transglycosylase D |
| P0.154 | *mdh* | Malate dehydrogenase |
| P0.156 | *mtfA* | Mlc titration factor A |
| P0.172 | *frdA* | Fumarate reductase ﬂavoprotein subunit |
| P0.185 | *pfkA* | Similar to E.coli 6-phosphofructokinase I |
| P0.212 | *pﬂE* | Putative pyruvate formate lyase activating enzyme |
| P0.271 | *nirB* | Nitrite reducatase large subunit |
| P0.272 | *ptsG* | PTS system, glucose-specific IIBC component |
| P0.301 | *glpA* | sn-glycerol-3-phosphate dehydrogenase subunit A |
| P0.310 | *ydiH* | Putative cytoplasmic protein |
