# Supplementary material for: Composing a Tumor Specific Bacterial Promoter
Source: PLoS One. 2016 May 12;11(5):e0155338. doi: 10.1371/journal.pone.0155338 (PMC4865170; doi:10.1371/journal.pone.0155338)
Supplement: S2 Table — Each module consists of two or more elements indicated by "+". P–values are calculated as a binomial probability to observe the actual number of promoters with a module in the TSP set compared to RP set. (DOC) [file pone.0155338.s005.doc]

**Table S2. Combinatorial modules found in tumor specific promoters.** Each module consists of two or more elements indicated by "+". P‑values are calculated as a binomial probability to observe the actual number of promoters with a module in the TSP set compared to RP set.

| Module | NagC | RscAB | FNR | MEF2 | TGIF | TEF | BRCZ4 | HNF1 | Meme1 | Meme2 | MDScan3 | MDScan5 | DME1 | A_8_ | Basal promoter | *P*-value |
| --- | --- | --- | --- | --- | --- | --- | --- | --- | --- | --- | --- | --- | --- | --- | --- | --- |
| 1 |  |  |  |  |  |  |  |  | **+** |  |  |  |  |  | **+** | 3.8*10^-23^ |
| 2 | **+** |  |  |  |  |  | **+** |  |  |  |  |  |  |  | **+** | 8.8*10^-14^ |
| 3 | **+** |  | **+** |  |  |  |  |  |  |  |  |  |  |  |  | 2.2*10^-20^ |
| 4 | **+** |  | **+** |  |  |  |  |  |  |  |  |  |  |  | **+** | 4.9*10^-21^ |
| 5 | **+** |  | **+** |  | **+** |  |  |  |  |  |  |  |  |  |  | 6.5*10^-19^ |
| 6 | **+** |  | **+** |  | **+** |  |  |  |  |  |  |  |  |  | **+** | 3.1*10^-21^ |
| 7 |  |  |  |  |  |  |  | **+** | **+** |  |  |  |  |  |  | 4.1*10^-19^ |
| 8 | **+** |  | **+** |  |  |  |  |  | **+** |  |  |  |  |  |  | 3.4*10^-23^ |
| 9 |  |  |  | **+** | **+** |  |  |  |  |  |  |  |  |  |  | 3.5*10^-14^ |
| 10 |  |  |  |  |  |  | **+** | **+** |  |  |  |  |  |  |  | 1.9*10^-09^ |
| 11 |  | **+** |  |  |  |  |  |  |  |  |  | **+** |  |  |  | 9.3*10^-13^ |
| 12 |  | **+** |  |  |  |  |  |  |  |  | **+** |  |  |  |  | 1.6*10^-13^ |
| 13 |  | **+** |  |  |  |  |  |  |  |  |  |  | **+** | **+** | **+** | 5.9*10^-19^ |
| 14 |  |  |  | **+** |  |  |  |  |  | **+** |  |  |  |  |  | 6.8*10^-15^ |
