# Supplementary material for: Composing a Tumor Specific Bacterial Promoter
Source: PLoS One. 2016 May 12;11(5):e0155338. doi: 10.1371/journal.pone.0155338 (PMC4865170; doi:10.1371/journal.pone.0155338)
Supplement: S1 Text — (DOC) [file pone.0155338.s006.doc]

**Text S1. DNA sequences of the tumor specific promoters.**

>P0.4

gatctttgatgaacagggacgtctgtgctgttcttcgcgtcttactacagcgattgtgtg

acgttgtcaccataaaagtgtgatgcatattgcttttggtcaaaatgtgcgcagaaaaag

gtgatatactgtgcacgtttacacataagcgaggatgatatgtctactgatttggaccca

acccaactggcgattgaatttttacgccgtgataaaaccgaactttctcccgcgcagtat

ttgaagcgtttaaaacagctggagttagagtttgccgatctcctcaccctctcagcaacc

gaactgaaagaagagatctatttcgcctggcggttgggcgtgcattaataaagtctgtgt

aggccggataaggcgttagccgccatccggctatgttaccaggcaatctcaccagacctt

gtccccaggtaatagataacccccttgattttgtttaaaaaaaatacagatc

>P0.48

gatcgatggcttcagcgccaatgcctgccgcttcacaggcgcgaataaaggtggcctgaa

acgcaagatcatcttccggcaacgtaataaacagaccatccgtcggttcgacgcaatggc

gggcgatccgttttaaaatctggttttcactaatgcactcgcgcgcggattccgcgtcgg

taaccgcataacgagcgccgctgtgcagcagcccgtggttacgcccggtcgcgccggtcg

ctatatcatgccgctccaccagaatgacacgtaaaccgcgcagcgcgcagtcgcgggcga

tccctgcgcctgttgctccaccgccaatgataatcacgtcacttgtttgcgagtcgcgag

ttttcattgtttttcctcacagttcgttttttatcatttagccatacaaatcatatgtaa

tgtttgatttcgcgcataatcgctcactattcgaaaatgaaacgtgatttcgtgcgcctt

tctgaacattagtcataaatctgtaacaatatgtgctgtaattcacattaacgtgacgta

tctttacttaacatcgcggccacactgggagcagcggggttgtttgaacgaactgcggtg

tttacctctaaataaaatatgggccacggaggctacaatatgttgagtatttttaaacca

gcgccgcataaagcgcgcttgccagcggcggagattgatccgacctatcgccgattacgc

tggcagattttcctggggatattctttggctatgccgcgtattatctggtgcgcaagaac

tttgccctcgcgatgccctacctggtagaacagggtttttcacgcggcgatctgggcttt

gcgctgtccgggatttccatcgcttatggtttttcgaaatttataatgggttcggtgtcc

gatcgctcgaatccgcgcgtttttctgccggcagggttgattctggccgcagcagtcatg

ttgtttatgggctttgtgccgtgggcgacatccagcatcgccgtgatgtttgtactgttg

ttcctttgcggctggttccaggggatggggtggccgccgtgcggtcgtacgatggttcac

tggtggtcgcagaaagagcgcggcggcattgtgtcggtctggaacggcgcgcataacgtc

ggcggcgggatc

>P0.92

gatcgctttacaggcgaacccccttctcaatcgtcatcggatgatttacggtacgttaac

ggcggaactttccacaaccgtacatgcgctggcactgcatacttataccctcaaagagtg

ggaaggattgcaggatactatcttcgcgtcgcctccttgtcgcggcgcgggaagcatcgc

gtagcaaaacggatttgcaactacctccgcttttccagtatgttgctacagaattatgtg

aaaacggcctgcgggccgttttgttttgtctgaattttgagcgtgtcgtacagtattcag

acaaaaattagccgagaattgtgaaaaccgccgcagcatcgcacaatcaccgttctcgac

tcacaaaagtgatgccgctataatgcgccgtcttatatatgaacgtcttcgggatgattc

tgacgacagggaatgtgattgattacgagaacatcccggttccgcgaagcaaatagcacg

tgcttgcggagtagagttgaccgagcactgtgattttttgaggtaacaagatgcaagttt

cagttgaaaccactcaggggccttggccgccgtgtaacgattacaatcgctgctgacagc

atcgagaccgctgtaaaaagcgagctggtcaacgtagcgaaaaaagtacgtattgacggc

ttccgtaaaggcaaagtaccgatgaatatcgtcgctcagcgttatggcgcttctgttcgc

caggacgtgctgggcgatcattccctggaacgccccgctttcacgctgcctgacgcttat

tgaaagcgtgcaggggcagcagttcagccgttacgtaccggaagacatcaccacgctact

gtcgatgacgcagccgttgaaactgcgcggttttcagccgtgggataccttctgcgatgc

catccatacgatgatgagcaacaccctgctccccgccgacgggaaaggcgttctggtcgc

gctgcgcccggtgccgggcattcgggttgagcaggcgttaacattatgtcggccaaacag

ggccggcgatattatgaccatcggcggcaaccgtctggaaaggtggttatcattctgccg

gggtctaacgatc

>P0.134

gatcccggcgaagcggcccccgtcttaaaggccattgctgaacataagtggatgccggag

gcgatttttttgacgcatcaccatcacgaccatgttggcggagtcaaagagctgttgcaa

cacttcccgcaaatgacggtttatggaccggcggaaacgcaagacaagggagcaacccat

cttgttggcgatggcgatactattcgcgttttaggcgagaaatttactctttttgccacg

ccgggccacacgttaggacacgtctgttactttagccgcccttacttattctgcggcgac

acgctgttttccggcggctgtggtcgactgtttgaaggcacgccatcacagatgtatcag

tcacttatgaaaattaactctctgcctgacgacacgctcatttgctgcgctcacgaatac

actttagctaacattaagttcgcattgagcatacttccgcacgattcgttcataaatgaa

tattatcgtaaagttaaagagttacgtgtaaaaaaacaaatgacattacccgttattctt

aaaaatgagcgtaagattaatctttttttaagaactgaagatattgatttaattaacgaa

ataaacaaagaaacaatattgcaacaaccagaagcgcgttttgcatggttaaggtcaaag

aaagacacgttctgataattcttacttgtcattcgctaacttcgccgttatgatc

>P0.154

gatccggattacgcaaattaaatgcataaaagccaaaattgcgcgactccgcattcttga

tgagtgaggattgtaatcattgaatttgtgaattaaggtcgccgccgcggagcaatagac

acttagctaatcatataataaggagtttaggatgaaagtcgcagtcctcggcgctgctgg

tggtatcggtcaggcgctggcattacttttaaaaaaccaactgccttcaggttcagaact

ctccctgtacgacatcgctccagtgactcccggtgtggccgttgatttgagccacatccc

caccgctgtaaaaatcaaaggtttctccggtgaagacgcaaccccggcgcttgaaggcgc

tgacgtagtactgatttctgcgggtgtggcgcgtaagccgggtatggaccgttccgacct

gtttaacgttaacgccggcatcgtgaaaaacctggtgcagcagatc

>P0.156

gatcgcaaggtgaaaatgagcccaaccctggacaggaagcgttgagcttttcgatgtgcg

ccagttaaaattctggcgtttttttctcaccgaattttctcattttttctcaacgtgatt

ttcatcactataagaaaatcacgtaagtgcttgaatagtggcggagagagagggattcga

accctcggcggagttacccccgcaacggttttcgagaccggtccgttcagccgctccggc

atctctccgtatattgcaatgatgccaggtaatttggcattttaacagaccctattcggg

taattttgttcaagtgacgagtttacgagcaaaacgatgattaagtggccctggaaagca

caagaaataacccagaacgaagactggccgtgggatgatgcgctggctatacctcttctg

gtaaacctcaccgcgcaagaacaggctcggcttattgcgctagccgaacgttttttgcag

cagaaaagactggtagcgctacagggatttgagctcgactcgttaaaaagtgcacgtatt

gcgttaattttttgcttaccgatc

>P0.172

gatcggcaaagaaacgacggatttccgccataatcgccgcacgttttaataaattgggga

tggacgcgctcggctgccaggttgccgtttcgctcatgattctttctccagtttaagaca

aggtcacgaagtctactcgcaacgcgcgggcgaaacaaattttgcgcaggcgtatcgggc

gccttctggagggtaaaaaaagtgatttcagatggtttagtaattaaattaatcaaaatc

aatgataattcatccctctgatacgctaaaaaaatcgaacacgtcaaatttccctcacat

ccctgagactatactgttgtacccataaaggagcagtggaaacgcattcatacttcgcag

aacccagaggctttatctggctgcgcgagggtgaaattacaataatctggaggaatgtcg

tgcaaacctttcaagccgatc

>P0.185

gatcgcgcgctgcccgatgcggaacgacaggaaattattgatatcgtgacgtcatggccg

ggagtcagcggcgcgcacgatctccgcacgcggcagtcagggccgactcgctttattcag

attcatttggaaatggaagataatctgccgctcgttcaggcgcattttgtggctgaccag

gtagagcaggcgattttacagcgttttccgggttcagatgtcattattcatcaggatccc

tgttcagtcgttcccagggaaggcaggaagttcgagcttgtataattgattgttaaaaag

tgagccaggccagcattttgtgtataaattaccgccatttggcctgacctgaatcaattc

agcaggaagggattgttatactatctgtatattcgttggatcgtttcgaagtgcgaaatc

ggcttccggcaatagatttcattttgcattccaaagttcagaggtagtcatgattaagaa

aatcggtgtgttgacaagcggcggtgatgcgccgggcatgaacgcggcaatccgcggtgt

tgtgcgcgcagcgttgacggaagggctggaagtcatgggcatttatgacggctatctggg

cctgtatgaagatc

>P0.212

gatcggttcgctacaggcaatggaggccattaagctactggcgcattacggtcagcctgc

cagcggaaaaatcgtcatgtacgatgcgatgacctgtcagttccgcgaaatgaagttaat

gcgcaaccccggctgtgaggtctgcgggcagtagccactttacggataaacataccaaag

cacggtttatggccgtgctgtatcaacagataattactgtcatcagacgcaaagccagct

tacccaagctccgactgacgatcggctcaagcagcgcggcatcatcttaaacgccatacc

acaacctcaaaccgtgatgttgtacctcatttaacgcctctttgtcagaacctctccatt

cgttgacgcacatcaagatagctttcattcgaaagtaatttaatctttatatgaaataag

agaggccgtttatgatc

>P0.271

gatcgcagcaaacgaaagtggcctcgcctaagctggttaacttcattctgacctgcggca

ccatcggaacaatgctgaccttcgtcgtcaccggcccgattgtagcgcacagcggcccac

aggcggcgttactcaccgcgaatggtctgtatgcggtggtctttgtgatgtgctttgcgc

tcggctttgtatcccgtcatcgtcagcatagcgcgccggctacgcattgataatccttgc

cggatggagacatcgccctccggcactctatcccccctcctgacggggtaggcctgttgg

tctaaaaacccctcattttgtatgttatttgtacaaacctgaaaagcctgacaattccgc

cacttataaaaatccagacaaatcagccatatacccattaagaggtatataaaggtgaat

ttgatttacatcaataagcggggttgctgaatcgttaaggtaggcggtaatagaaaagaa

atcgaggcaaaaatgagcaaagtcagactcgctattatcggtaatggtatggtcggccac

cgctttattgaggatc

>P0.272

gatcaccaccgataactttgctcgcctgttccatattgacgcgtctcgcctgtcatcaat

ccgttaaacgagtttttttaaagctcgtaattaataaacaaaacgcgtaaagttcaccgc

cacaaaaggggcggtgagcgagcttatggaaacattcggaaactcattttggcagaatgt

gatactttttaggctatctggcgctgaaacgtgatagccgtcaaacaaaatcagacgtat

ttattttactctgtgtaataaataaaagggcacttagatgtcctgtccacggcggggttc

tcccccctcgccaatgcgtgagaacgtagaaaagcacaaatactcaggagcactctcaat

tatgtttaagaatgcatttgctaacctgcaaaaggtcggtaaatcgctgatgctgccggt

atccgtactccctatcgcaggtatcctgctgggtgtcggttccgctaacttcagctggct

gccagccgttgtatcgcacgttatggcagaagcgggcggatc

>P0.301

gatcgccgcgtgaaaaaccctgttctaccaggtagggcatcgcgagggcaaagttcttgc

gcaccagataatacgcggcatagccaaagaatatccccaggaaaatctgccagcgtaatc

ggcgataggtcggatcaatctccgccgctggcaagcgcgctttatgcggcgctggtttaa

aaatactcaacatattgtagcctccgtggcccatattttatttagaggtaaacaccgcag

ttcgttcaaacaaccccgctgctcccagtgtggccgcgatgttaagtaaagatacgtcac

gttaatgtgaattacagcacatattgttacagatttatgactaatgttcagaaaggcgca

cgaaatcacgtttcattttcgaatagtgagcgattatgcgcgaaatcaaacattacatat

gatttgtatggctaaatgataaaaaacgaactgtgaggaaaaacaatgaaaactcgcgac

tcgcaaacaagtgacgtgattatcattggcggtggagcaacaggcgcagggatcgcccgc

gactgcgcgctgcgcggtttacgtgtcattctggtggagcggcatgatatagcgaccggc

gcgaccgggcgtaaccacgggctgctgcacagcggcgctcgttatgcggttaccgacgcg

gaatccgcgcgcgagtgcattagtgaaaaccagattttaaaacggatc

>P0.310

gatcgtatctttttataaaacacaaccattttttataaacatcctgattgaaattgtcat

aaactattacccggagttttggagtccagcaaccaaaggagacggaatgcatcacgctac

cccgcttatcaccaccattgtcggcggccttgtgctcgcttttattctcggcatgattgc

caacaaattgcgtatttctccactggtgggatatctgttagcgggcgttctggcgggacc

ttttaccccgggttttgttgcggataccaaactggcgccggagctggcggagctcggcgt

gattctattgatgttcggcgtcgggctgcatttttcgctgaaggatttgatggcggtaaa

gtctatcgccattcccggcgctgtcgctcagatagcggtggcgacgctgctgggtatggc

gctttccgccgtgctgggatggtcattaatgaccggcatcgtttttgggctctgtctgtc

tacagccagtaccgtcgtcctgctgcgcgcgcttgaggagcgacaactccttgatagcca

gcgcgggcaaatcgccatcggctggctgattgtcgaagatc
